# Coordination templated [2+2+2] cyclotrimerization in a porous coordination framework

**DOI:** 10.1038/ncomms9348

**Published:** 2015-09-18

**Authors:** Yong-Sheng Wei, Mei Zhang, Pei-Qin Liao, Rui-Biao Lin, Tai-Yang Li, Guang Shao, Jie-Peng Zhang, Xiao-Ming Chen

**Affiliations:** 1 MOE Key Laboratory of Bioinorganic and Synthetic Chemistry, School of Chemistry & Chemical Engineering, Sun Yat-Sen University, Guangzhou 510275, China

## Abstract

Controlling chemical reactions by the supramolecular confinement effects of nanopores has attracted great attention. Here we show that open metal sites in porous coordination frameworks can constitute more powerful and strict templates for precision syntheses. A Fe(III) dicarboxylate framework functionalized with triangularly arranged metal sites is used to accomplish [2+2+2] cyclotrimerization reactions for organonitrile, alkyne and alkene monomers bearing a geometrically suitable pyridyl group. *In situ* single-crystal X-ray diffraction facilitates the direct observation of such a coordination templated reaction, before cylcotrimerization, the monomer coordinates at the Fe(III) centre by its pyridyl donor, which forces three unsaturated groups to gather around a position very similar with that of the desired covalent cyclic trimer. After the reaction, the trimers serve as tripodal ligands to perfectly fix the Fe(III) ions and the whole crystal to generate an exceptionally rigid and porous material with large surface area coupled with guest-proof zero thermal expansion.

## Abstract (web summary)

Nanospace defined by unsaturated metal coordination sites opens up the possibility of controlling and templating chemical reactivity. Here, the authors show that a Fe(III) dicarboxylate framework with triangularly arranged metal sites can direct the [2+2+2] cyclisation of nitriles, alkynes and alkenes.

## Graphical abstract

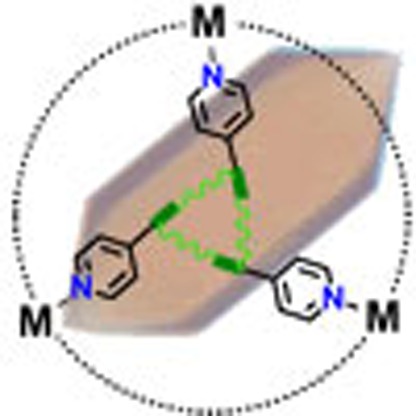

Besides many useful applications such as storage, separation, catalysis, and so on, porous materials may also serve as nano-reactors to control the molecular structures and morphologies of the products by restricting the space around the guest reactants/products, and even form host-guest nanocomposites with interesting properties^1^,^2^,^3^. Compared with the conventional bulk reactions, the structrues, molecular weights and stereoregularity of polymer products obtained in porous hosts^4^, especially periodic/crystalline ones with microporous channels^5^,^6^,^7^, can be regulated to much higher levels. When the reactant and products are both small molecules, organic reactions such as Diels-Alder addition^8^, isomerization^9^ and photodimerization^10^ can be more rationally controlled^11^, because the guest molecules can fit the periodicity of the pore environment to enable good host-guest matching^12^. More importantly, some reaction stages may be directly visualized by single-crystal X-ray diffraction (SCXRD)^11^,^13^,^14^, although keeping the sample single-crystallinity after physical/chemical changes is always a great challenge^15^,^16^,^17^,^18^,^19^,^20^,^21^,^22^,^23^.

Because the pore and the guest recognize each other by hydrogen bonding, π–π stacking, and more commonly other weaker supramolecular interactions, their sizes and shapes must be commensurate so that the guest reactants can be fixed at the right positions to achieve good control over the chemical reaction. In other words, the host always loses porosity after carrying out such precision chemical syntheses. On the other hand, by virtue of the strong and highly directional coordination bonds, metal ions and organic bridging ligands can self-assemble to build crystalline open frameworks (well known as porous coordination polymers or metal-organic frameworks) with overwhelmingly high porosity and robustness compared with other molecular materials^24^,^25^,^26^,^27^,^28^,^29^. In many cases, the coordination sites of the metal ions are not fully used by the bridging ligands. The unoccupied or open metal sites can bind guest molecules strongly at specific positions/directions, which should be ideal for development of strictly controlled reactors/reactions. Herein we prove this concept by using a prototypical material [Fe_3_(*μ*_3_-O)(bdc)_3_X(H_2_O)_2_] (MIL-88B (refs 30, 31, 32); H_2_bdc=1,4-benzenedicarboxylic acid, X=OH^−^/Cl^−^/F^−^) as the host coordination template. A series of facile and strictly selective [2+2+2] coordination templated cyclotrimerization (CTC) reactions have been realized. Unlike other molecular reactors, the reaction can drastically improve the gas adsorption performance of the host crystal.

## Results

### Synthesis and structure

**Figure 1:**
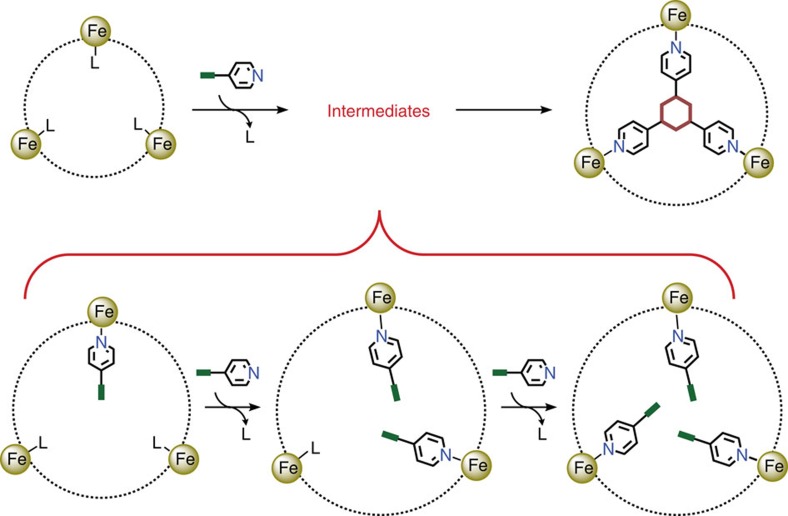
The proposed CTC reaction mechanism. The monomer molecule bearing an unsaturated functional group (thick green stick) and an anchor (the pyridyl group) replaces the terminal ligand (L) originally coordinated at the Fe(III) site. The convergent and triangular arrangement of the Fe(III) sites forces every three unsaturated groups of the monomer ligands to gather in a close configuration suitable for the cyclotrimerization reaction. The ligand replacement occurs at a step-by-step manner.

MIL-88B is well known for its highly flexible behaviours towards various organic molecules^30^,^31^,^32^. An interesting structural feature of MIL-88B is that every three adjacent Fe(III) ions locating at the same channel (from three Fe_3_(*μ*_3_-O) clusters) defines a regular triangle with converging Fe-H_2_O/X vectors. The highly directional coordination bonds of the octahedral Fe(III) ions with such an alignment may fix three molecules in an ideal configuration to facilitate the [2+2+2] CTC reactions (Fig. 1). Considering the geometry of such a Fe_3_ coordination template, we selected 4-cyanopyridine (4-pyCN), 4-ethynylpyridine (4-pyC_2_H) and 4-vinylpyridine (4-pyC_2_H_3_), each bearing a coordination donor at one end and an unsaturated group at the opposite end, as the reactants or monomers.

The CTC reactions were accomplished by loading the monomers in microcrystalline MIL-88B, removing excess monomers outside the microcrystal host, and then heating at 140 °C within 12 h, or simply heating a mixture of MIL-88B and excess monomers at the same condition. Powder X-ray diffraction (PXRD) showed that, the unit-cell volumes of the resultant crystalline products are even larger than that of the most-open form of MIL-88B (Supplementary Figs 2 and 3)^31^,^32^. The desired trisubstituted *s*-triazine, benzene, and cyclohexane derivatives, namely 2,4,6-tri(pyridin-4-yl)-1,3,5-triazine (tpt), 1,3,5-tri(pyridin-4-yl)benzene (tpb) and 1,3,5-tri(pyridin-4-yl)cyclohexane (tpc), were first detected by infrared (IR) spectroscopy and electrospray ionization mass spectrometry (ESI-MS) analyses of the resultant crystalline products (Supplementary Figs 4 and 5). The formation of these cyclic trimers inside the Fe_3_ coordination templates was further confirmed by SCXRD. It should be noted that, MIL-88B has been always synthesized as microcrystalline powders^30^,^33^, but we now succeeded to grow large single crystals by using hydrofluoric acid to replace the conventionally used NaOH reactant. Finally, the organic products were recovered from the acid-digested crystalline products and fully characterized by IR, ESI-MS, ^1^H and ^13^C nuclear magnetic resonance (NMR) spectroscopy (Supplementary Figs 6-11).

**Figure 2:**
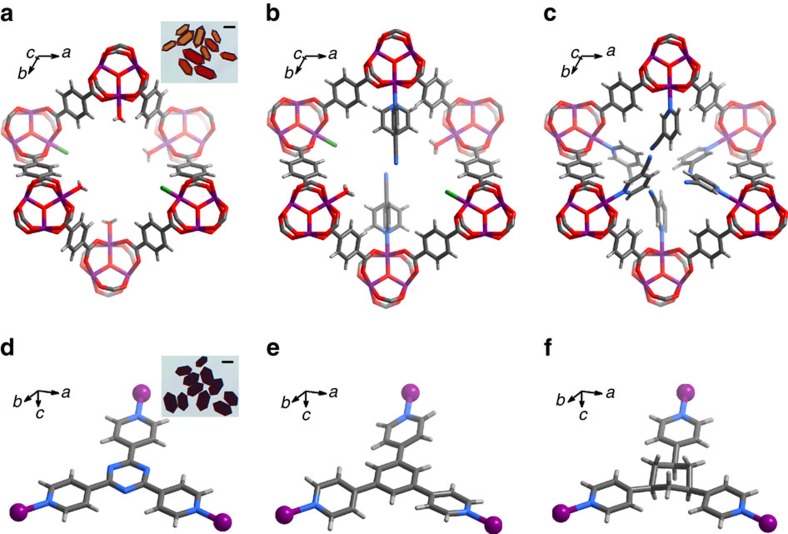
X-ray single-crystal structures. (**a**) MIL-88B. (**b**) 4-pyCN@MIL-88B. (**c**) 3-pyCN@MIL-88B. (**d**–**f**) The trimers tpt, tpb and tpc embedded in MIL-88B-tpt, MIL-88B-tpb and MIL-88B-tpc, respectively. Color codes: dark grey, C; light grey, H; blue, N; red, O; purple, Fe; green, F. The pyridyl ring of 4-pyCN is two-fold disordered at conformations parallel and perpendicular with the Fe_3_ plane. Some other crystal structures are symmetrically disordered, only one possible structure is shown for clarity. Inset: photographs of corresponding compounds (Scale bars, 0.10 mm).

On the basis of the SCXRD data, the chemical formulae of the product crystals obtained by the CTC reactions are determined as [Fe_3_(*μ*_3_-O)(bdc)_3_(tpt)]X (MIL-88B-tpt), [Fe_3_(*μ*_3_-O)(bdc)_3_(tpb)]X (MIL-88B-tpb) and [Fe_3_(*μ*_3_-O)(bdc)_3_(tpc)]X (MIL-88B-tpc), respectively, (Fig. 2 and Supplementary Table 1). Besides the original 6-connected **acs** networks of [Fe_3_(*μ*_3_-O)(bdc)_3_], each Fe_3_ coordination template embeds a tpt/tpb/tpc ligand, which coordinates with the three Fe(III) ions by its three pyridyl ends (Fig. 2 and Supplementary Fig. 1). The coordination networks of the product crystals possess a rare binodal (3,9)-connected **nia-d** topology^34^, regarding the tpt/tpb/tpc ligands and the Fe_3_ clusters as 3- and 9-connected nodes, respectively. Interestingly, the unit-cell volumes of the product crystals are ca. 145, 18 and 9% larger than those of the dry, as-synthesized, and most-open forms of MIL-88B, respectively^31^, because the large tpt/tpb/tpc ligands force the host framework to expand to an extent not possible for lattice guest molecules.

### Reaction mechanism

**Figure 3:**
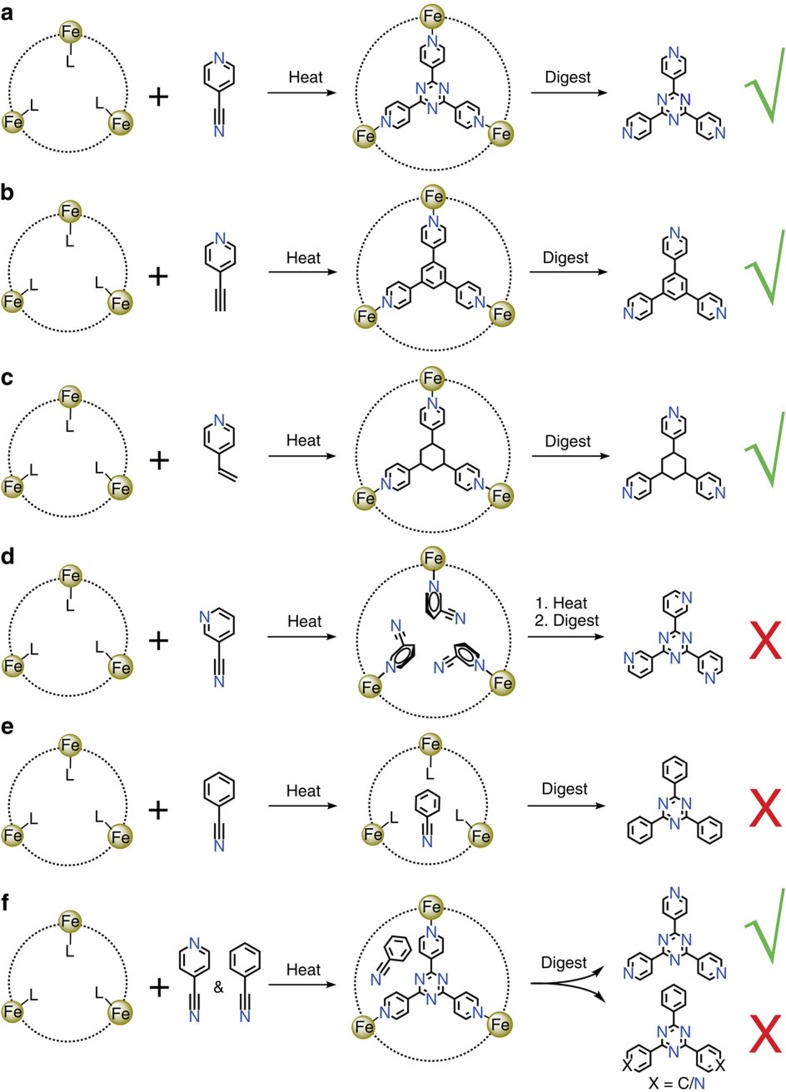
Summary of the CTC reactions for various monomers. (**a–c**) When 4-pyCN, 4-pyC_2_H, and 4-pyC_2_H_3_ were used as monomers, the corresponding trimeric products were observed by ESI-MS, IR, ^1^H NMR, ^13^C NMR and SCXRD. (**d**) When 3-pyCN was used as the monomer, the coordination of 3-pyCN molecules on the Fe(III) centre was observed by SCXRD, but the expected trimeric product could not be detected by ESI-MS, IR or SCXRD. (**e**) When PhCN was used as the monomer, the expected trimeric product was not detected by ESI-MS, IR or SCXRD. (**f**) When a mixture of 4-pyCN and PhCN was used as monomers, only tpt was detected by ESI-MS among four possible trimeric products.

To verify the CTC reaction mechanism, control experiments were performed. Using 3-cyanopyridine (3-pyCN) and benzonitrile (PhCN) as monomers (Fig. 3d,e), the trimeric product of 3-pyCN/PhCN was not detected by either IR or ESI-MS of the reaction mixtures (Supplementary Figs 12 and 13). When a mixture of 4-pyCN and PhCN was used to perform the same reaction, only tpt was detected among four possible trimeric products (Fig. 3f and Supplementary Fig. 14). These results confirmed that MIL-88B is not able to catalyse the trimerization reactions involving monomers with unsuitable coordination sites and molecular geometry. More importantly, when excess 4-pyCN monomers were used for the CTC reaction, tpt cannot be detected out of the Fe_3_ coordination templates (Supplementary Fig. 15), meaning that in MIL-88B-tpt, the trimeric product tpt is solely generated at the Fe_3_ coordination templates. Note that the CTC reactions can be furnished in very high yields (>90%) without using excess monomers (Supplementary Figs 3–5), confirming the rule of the Fe_3_ coordination template and exclude the possibilities of cyclotrimerization at other sites such as a single metal centre locating at defects and/or outer surface of the crystals.

To reveal more details for the CTC reaction mechanism, we carried out an *in situ* SCXRD study. The unit-cell volume of the crystal stabilized at a value of 16% larger than that of as-synthesized MIL-88B but 1.0% smaller than MIL-88B-tpt, after heated to 110 °C for 24 h. Structure analysis revealed a stable composition of [Fe_3_(*μ*_3_-O)(bdc)_3_(4-pyCN)X(H_2_O)] (denoted as 4-pyCN@MIL-88B, Supplementary Table 1), indicating that one third of terminal H_2_O/X ligands are substituted by 4-pyCN molecules. From the crystal structure (Fig. 2b), it can be clearly seen that 4-pyCN coordinates at the Fe(III) ion using its pyridyl N donor, so that its cyano group is pointed at the centre of the Fe_3_ coordination template. The pyridyl ring of 4-pyCN in 4-pyCN@MIL-88B showed obvious conformational disorder, because the pyridyl C-H moieties has no specific interaction with other parts. In contrast, the pyridyl and triazine rings of tpt in MIL-88B-tpt are strictly coplanar (Fig. 2d). Further increasing the heating time and/or temperature resulted in monotonic and small increase of the unit-cell volume until a stable value virtually identical with that of MIL-88B-tpt, which was not changed after heated at 180 °C for 24 h. As shown in Supplementary Fig. 16, similar phenomena can be also observed by PXRD. Structure analysis confirmed that the crystal at this stage is identical with MIL-88B-tpt (Supplementary Table 1), meaning that the cyclotrimerization reaction was completed.

For comparison, 3-pyCN was also used for the *in situ* SCXRD study. Structure analysis of the crystal above the melting point of 3-pyCN revealed a chemical formula of [Fe_3_(*μ*_3_-O)(bdc)_3_(3-pyCN)_3_]X (denoted as 3-pyCN@MIL-88B, Supplementary Table 1), meaning that each Fe(III) ion is coordinated by one 3-pyCN, or all terminal H_2_O/X ligands are completely substituted. Because the cyano group locates at the meta-position of the pyridyl ring, there is no steric hindrance among three 3-pyCN ligands in the same Fe_3_ coordination template (Fig. 2c). Unlike 4-pyCN, the pyridyl ring of 3-pyCN is almost perpendicular with the Fe_3_ plane, because with this conformation the cyano group can form weak hydrogen bonds (N^…^C 3.81(4) Å) with the pyridyl ring of a neighbour locating at another Fe_3_ coordination template (Supplementary Fig. 17). In other words, the cyano groups of 3-pyCN monomers are evenly distributed in 3-pyCN@MIL-88B and fixed at positions distinctly unfit for the trimerization reaction. Consequently, the corresponding triazine product of 3-pyCN cannot be obtained by further increasing the heating time/temperature.

### Modification of flexibility and porosity

**Figure 4:**
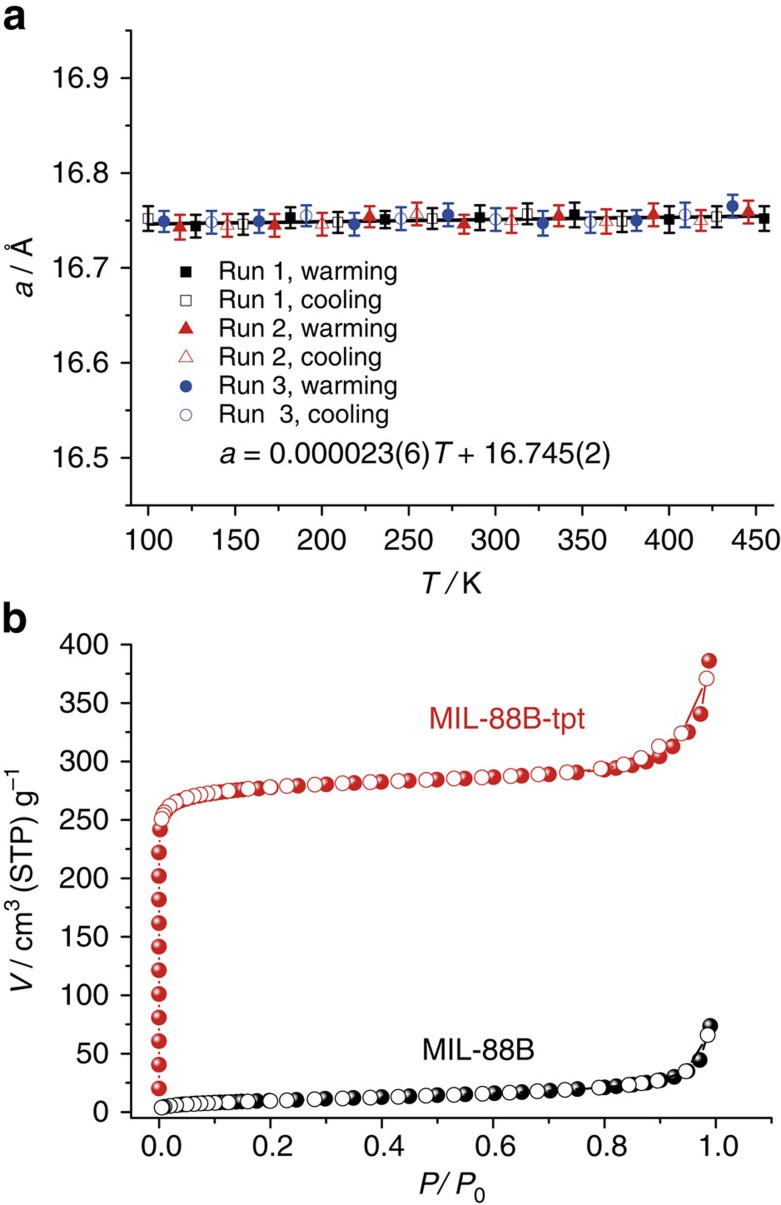
Framework flexibility and gas adsorption property of the host-guest composite. (**a**) Temperature- and guest-dependences of the *a*-axis length of MIL-88B-tpt obtained in several heating/cooling cycles. The as-synthesized crystal of MIL-88B-tpt was used to start the measurement. Obviously, the *a*-axis length is nearly unaffected by guest and temperature, giving zero thermal expansion across the *ab*-plane. (**b**) N_2_ adsorption (filled) and desorption (open) isotherm of MIL-88B and MIL-88B-tpt.

Due to the additional tripodal ligands, the product crystals obtained by the CTC reactions display very different framework flexibility and adsorption property compared with MIL-88B. Thermogravimetry analyses and variable-temperature PXRD showed that MIL-88B-tpt is a rigid framework stable up to 400 °C (Supplementary Figs 18 and 19). Variable-temperature SCXRD at 100–455 K showed that guest and temperature can barely affect the unit-cell parameters of MIL-88B-tpt, giving zero thermal expansion across the *ab*-plane and very small negative thermal expansion along the *c*-axis (Fig. 4a and Supplementary Figs 19b and 20), which is exceptional for such a porous structure^35^,^36^,^37^. Further, guest-free MIL-88B-tpt showed a type-I N_2_ isotherm, corresponding with a Brunauer–Emmett–Teller surface area of 1,113 m^2^ g^−1^ and a Langmuir surface area of 1,331 m^2^ g^−1^, as well as a pore volume of 0.56 cm^3^ g^−1^. In contrast, guest-free MIL-88B showed a type-II N_2_ isotherm with very small uptake, surface area, and pore volume (Fig. 4b), because the shrunk coordination framework of MIL-88B can be hardly opened by N_2_ molecules^38^, although it can undergo significant solvent-induced breathing.

## Discussion

In conventional approaches, cyano, alkyne and alkene groups are quite different in reactivity. Cyclotrimerization of organonitriles to form 1,3,5-triazine derivatives is stereo selective, but both catalyst and high reaction temperature (>200 °C) are required^39^,^40^. The synthesis of benzene derivatives through catalysed cyclotrimerization of alkynes, which usually forms 1,2,4- and 1,3,5-trisubstituted isomers as a mixture, concomitant with polymers as byproducts, has been studied extensively^41^. Without catalyst, alkenes can easily polymerize to form long chains. On the other hand, when two alkene molecules are properly aligned (usually in a crystal) with separation less than 4.1 Å, photo/thermochemical [2+2] cycloaddition can occur to generate cyclobutane with high stereo selectivity^9^,^15^. Nevertheless, by using weak supramolecular interactions and pore confinement effects, three alkene molecules can be hardly fixed in a configuration suitable for [2+2+2] cyclotrimerization, so that cyclohexanes are generally obtained by hydrogenation of benzenes. Obviously, MIL-88B greatly facilitates [2+2+2] cyclotrimerization reactions of organonitrile, alkyne, and alkene monomers bearing a geometrically suitable pyridyl group, highlighting the coordination templating effects of its open metal sites.

The results of *in situ* SCXRD showed that the intermediate state with a formula of [Fe_3_(**μ**_3_-O)(bdc)_3_(4-pyCN)_3_]X, that is, three 4-pyCN monomers simultaneously coordinate at the same Fe_3_ coordination template, cannot be observed by diffraction techniques that are suitable for characterizing thermodynamic equilibrium structures^15^,^16^,^17^,^18^,^19^. Obviously, the supramolecular trimer (4-pyCN)_3_ has a larger size compared with the covalent trimer tpt, meaning that the unit-cell volume of [Fe_3_(*μ*_3_-O)(bdc)_3_(4-pyCN)_3_]X needs to be larger than that of MIL-88B-tpt. However, the unit-cell volume of the most-open form of MIL-88B, obtained by inclusion of large organic molecules^31^,^32^, is still smaller than that of MIL-88B-tpt, indicating that [Fe_3_(*μ*_3_-O)(bdc)_3_(4-pyCN)_3_]X with an even larger unit-cell volume should be energetically unfavourable, at least at the thermodynamic equilibrium state. In other words, such a theoretical intermediate state may be too reactive, which occurs transiently and transforms to the trimeric product immediately. Therefore, the CTC reaction might be mainly controlled by the ligand substitution reaction rather than the organic reaction. Indeed, it has been demonstrated that other organic molecules, including pyridine (with a stronger coordination ability compared with 4-pyCN), always serve as pure lattice guests, and cannot substitute the H_2_O/X ligand in all MIL-88 series at room temperature^31^,^32^. On the other hand, while there is no conventional catalytic site in MIL-88B, the 4-pyCN to tpt transformation occurs at a relatively low temperature, which implies that the flexible coordination network and the Fe_3_ coordination template, having a large tendency to shrink at the transient intermediate state [Fe_3_(*μ*_3_-O)(bdc)_3_(4-pyCN)_3_]X, might compress the supramolecular trimer (4-pyCN)_3_ and force it to form the covalent trimer tpt.

In summary, we demonstrated that with a judicious geometric consideration of the host-guest structure, the open metal sites in a porous coordination framework can be rationally used to fix guest molecules to facilitate chemical reaction difficult or even impossible for conventional methods and other nano-reactors. Importantly, the template crystal can retain single-crystallinity throughout the supramolecular and molecular reaction processes, allowing the direct observation of several key reaction stages. Also interestingly, the precisely controlled reaction can drastically improve the framework rigidity and gas adsorption performance of the crystalline host. These results should be instructive for further exploration of new chemical reactions and functions using porous crystals as a platform.

## Methods

### Materials and physical measurements

Reagents were commercially available and used without further purification except otherwise stated. The monomer 4-pyC_2_H_3_ was distilled to remove polymerization inhibitor hydroquinone before use. ESI-MS was obtained using a Shimadzu LCMS-2010A mass spectrometer, for which samples were digested by sonication in a mixture of hydrochloric acid and MeOH after removal of the residual monomers by sublimation under vacuum. Elemental analyses (EA) were conducted using an Elementar Vario EL analyzer using guest-free samples (with a little water adsorbed from air). IR spectra were recorded on a Bruker TENSOR 27 FT-IR spectrometer in the 400–4,000 cm^−1^ region with KBr pellets. ^1^H NMR and ^13^C NMR spectra were recorded on a Bruker AVANCE III 400 MHz NMR spectrometer. Chemical shifts were quoted in p.p.m. referenced to the appropriate solvent peak or 0.0 p.p.m. for tetramethylsilicane. PXRD patterns were collected on a Bruker D8-Advance diffractometer with Cu Kα radiation and a LynxEye detector. Thermogravimetry analyses were performed on a TA Q50 system under N_2_ at a heating rate of 5 °C min. N_2_ sorption isotherms were measured by a Micromeritics ASAP 2020M physisorption analyzer.

### Synthesis of MIL-88B

Microcrystals^30^,^33^: A mixture of FeCl_3_·6H_2_O (4.32 g, 16.0 mmol) and H_2_bdc (2.66 g, 16.0 mmol) were dispersed in *N,N*-dimethylformamide (DMF; 80.0 ml) with 2 M NaOH (6.4 ml). The mixture was placed in a 100-ml vial and sealed with a screw cap, which was heated at 100 °C for 12 h. Then, the orange solid was filtered, washed with DMF, and dried in air overnight to give [Fe_3_(*μ*_3_-O)Cl(H_2_O)_2_(bdc)_3_]·guest (yield 60%). The content of chloride ion was determined by potentiometric titration with the acid-digested samples. Large single crystals: a mixture of FeCl_3_·6H_2_O (0.081 g, 0.3 mmol), H_2_bdc (0.050 g, 0.3 mmol), hydrofluoric acid (0.1 ml, 49 wt% in H_2_O), and DMF (3.0 ml) was stirred for 30 min in air, then transferred and sealed in a 20-ml vial with a screw cap, which was heated at 90 °C for 3 days and then cooled to room temperature, giving orange block crystals, [Fe_3_(*μ*_3_-O)F_0.74_(OH)_0.26_(H_2_O)_2_(bdc)_3_]·guest (yield 50%). The content of fluoride ion was determined by the fluoride selective electrode with the acid-digested samples.

### Coordination templated cyclotrimerization reactions

The as-synthesized microcrystals of MIL-88B were exchanged with hot MeOH using a Soxhlet extractor to remove the unreacted FeCl_3_ and H_2_bdc in the channels. The reaction method without using excess monomers: the monomer was loaded into MIL-88B (0.100 g) by mixing it with 4-pyCN (1.00 g) and heated at 110 °C for 24 h, immersing it in an anhydrous toluene solution (20.0 mL) of 4-pyC_2_H (0.100 g) and heated at 50 °C for 6 h under N_2_, or by immersing it in 4-pyC_2_H_3_ (1.0 ml) at room temperature for 3 days; After removal of the excess monomers by sublimation for 4-pyCN or washing with CH_2_Cl_2_ for 4-pyC_2_H and 4-pyC_2_H_3_, the monomer-loaded MIL-88B was transferred and sealed in a 1-ml glass tube, heated in an oven at 140 °C for 12 h and then cooled to room temperature, giving black products (yields: 92%, 94% and 91%, respectively). The reaction method using excess monomers: a mixture of MIL-88B (0.100 g) and the monomer, that is, 4-pyCN (3.00 g) or DMF (3.0 ml) solution of 4-pyC_2_H (0.140 g) or 4-pyC_2_H_3_ (3.0 ml) sealed in a 25-ml teflon-lined stainless container, and the products were finally washed by DMF and dried in air (yield>90%). MIL-88B-tpt: EA calc. (%) for [Fe_3_(*μ*_3_-O)(bdc)_3_(tpt)]Cl·9H_2_O (C_42_H_42_ClFe_3_N_6_O_22_): C, 43.14; H, 3.62; N, 7.19; found: C, 44.09; H, 3.92; N, 7.03. MIL-88B-tpb: EA calc. (%) for [Fe_3_(*μ*_3_-O)(bdc)_3_(tpb)]Cl·14H_2_O (C_45_H_55_ClFe_3_N_3_O_27_): C, 42.46; H, 4.36; N, 3.30; found: C, 42.41; H, 4.51; N, 3.30. MIL-88B-tpc: EA calc. (%) for [Fe_3_(*μ*_3_-O)(bdc)_3_(tpc)]Cl·7H_2_O (C_45_H_47_ClFe_3_N_3_O_20_): C, 46.88; H, 4.11; N, 3.64; found: C, 47.41; H, 4.24; N, 3.57. For samples used for SCXRD studies, the as-synthesized single crystals of MIL-88B were directly used, and the reactions were carried out at 180 °C for 24 h.

### Separation of covalent trimers

The host-guest hybrid crystals were firstly dissolved in concentrated hydrochloric acid with stirring for 20 min. After removing the undissolved white solid (H_2_bdc) by filtration, pH of the solution was adjusted to ∼11 with aqueous NaOH (1 M). After that, the aqueous solution was extracted with CH_2_Cl_2_ for three times. The combined organic phase was dried over anhydrous Mg_2_SO_4_ and concentrated to remove solvent under reduced pressure, finally giving white products.

### Single-crystal X-ray diffraction analyses

Single-crystal X-ray diffraction data were collected on an Oxford Gemini-S Ultra CCD-detector or a Rigaku XtaLAB P300DS-detector diffractometer (Cu Kα). The measurement temperatures were controlled by a dry nitrogen flow using a Rigaku Gas Flow GN2 apparatus. For the *in situ* SCXRD study, a single crystal of MIL-88B and excess amount of monomer were sealed in a glass capillary. Nevertheless, identical single-crystal structure can be also obtained without using excess monomer. The diffraction data were collected just above the melting point of the monomer to avoid the interference of diffraction of crystalline monomer. All structures were solved by the direct method and refined with the full-matrix least-squares technique on *F*^2^ by the *SHELXTL* software package. The terminal coordinated molecules/anions, namely, H_2_O, OH^–^ and F^–^ in MIL-88B, H_2_O, OH^–^, F^–^ and 4-pyCN in 4-pyCN@MIL-88B, are statistically and symmetrically disordered at the three terminal sites (crystallography three-fold symmetry) of one Fe_3_(**μ**_3_-O)(bdc)_3_(L^T^)_3_ (L^T^=terminal ligand). Since the donor atoms occupy essentially the same positions and cannot be distinguished by X-ray diffraction, they were refined using the EADP and EXYZ constraints. In 4-pyCN@MIL-88B and MIL-88B-tpc, the pyridyl rings of 4-pyCN and tpc are statistically/dynamically two-fold disordered at conformations parallel and perpendicular with the Fe_3_ plane. In MIL-88B-tpb, the pyridyl rings of tpb are statistically/dynamically/symmetrically two-fold disordered with molecular planes about 18^o^ with the Fe_3_ plane. In 3-pyCN@MIL-88B, the coordinated 3-pyCN molecule is symmetrically four-fold disordered because the bent molecule is locates at a two-fold axis and a mirror plane. Moreover, in MIL-88B-tpc, the non-planar cyclohexane ring is in the *e*,*e*,*e*-conformation and symmetrically two-fold disordered at the crystallography mirror plane. The SHELXTL restraint instructions DFIX, SIMU, ISOR and FLAT were applied to these disordered groups to keep their geometries and atomic displacement parameters reasonable. All host-framework non-hydrogen atoms were refined anisotropically. Hydrogen atoms were placed geometrically. The PLATON SQUEEZE treatment was applied because all lattice guests are extremely disordered and cannot be modelled. Detailed structure determination parameters and crystallographic data are given in Supplementary Table 1.

## Additional information

**Accession codes.** The X-ray crystallographic coordinates for structures reported in this study have been deposited at the Cambridge Crystallographic Data Centre (CCDC), under deposition numbers 1415803-1415810. These data can be obtained free of charge from The Cambridge Crystallographic Data Centre via www.ccdc.cam.ac.uk/data_request/cif.

**How to cite this article:** Wei, Y.-S. *et al.* Coordination templated [2+2+2] cyclotrimerization in a porous coordination framework. *Nat. Commun.* 6:8348 doi: 10.1038/ncomms9348 (2015).

## Supplementary Material

Supplementary Information Supplementary Figures 1-20, Supplementary Tables 1-2 and Supplementary Methods
