# Supplementary material for: Coordination templated [2+2+2] cyclotrimerization in a porous coordination framework
Source: Nat Commun. 2015 Sep 18;6:8348. doi: 10.1038/ncomms9348 (PMC4595715; doi:10.1038/ncomms9348)
Supplement: Supplementary Information — Supplementary Figures 1-20, Supplementary Tables 1-2 and Supplementary Methods [file ncomms9348-s1.pdf]

(a)

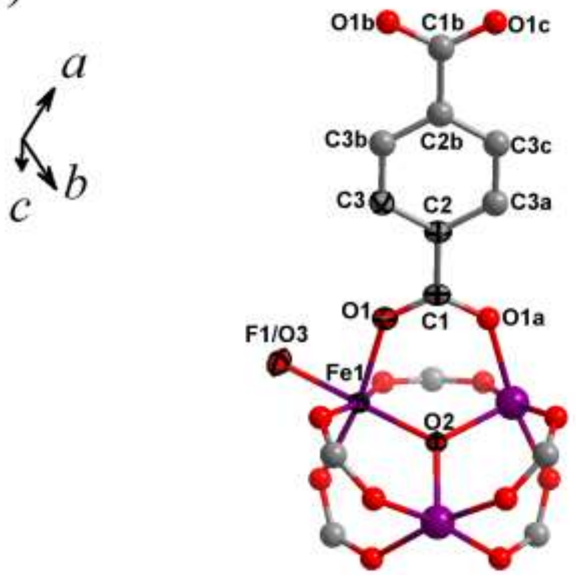

Supplementary Figure 1a. Perspective views of the coordination environments in MIL-88B. Hydrogen atoms are omitted for clarity (Thermal ellipsoids are drawn for the asymmetric units with probability 30%). Symmetry codes: a = 1-*y*, 1-*x*, *z*; b = *y*, *x*, 1-z; c = 1-*x*, 1-*y*, 1-z.

(b)

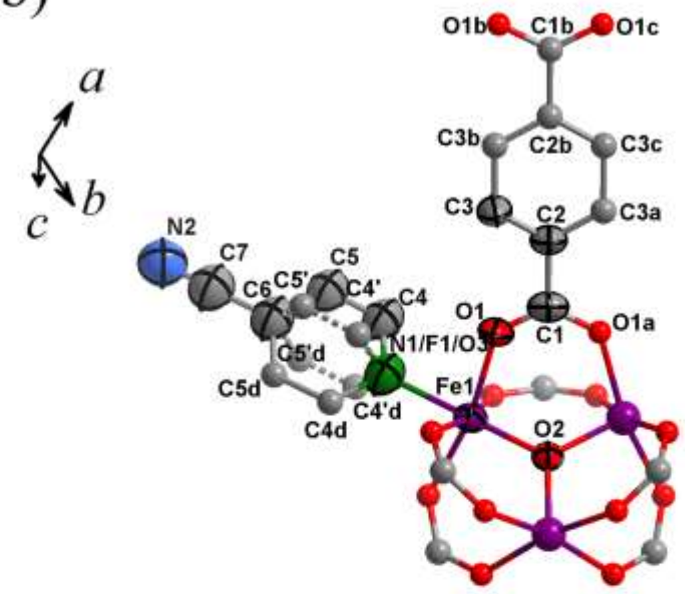

Supplementary Figure 1b. Perspective views of the coordination environments in 4-pyCN@MIL-88B. Hydrogen atoms are omitted for clarity (Thermal ellipsoids are drawn for the asymmetric units with probability 30%). Dashed bonds represent another part of the 2-fold disordered pyridyl group. Symmetry codes: a = 1-*y*, 1-*x*, *z*; b = *y*, *x*, 1-*z*; c = 1-*x*, 1-*y*, 1-*z*; d = -*x*+*y*, *y*, 1.5-z.

(c)

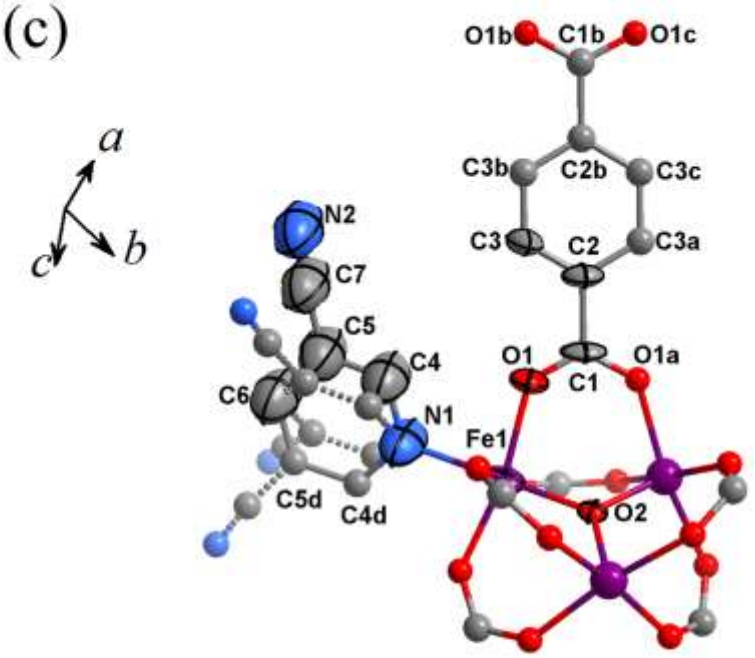

Supplementary Figure 1c. Perspective views of the coordination environments in 3-pyCN@MIL-88B. Hydrogen atoms are omitted for clarity (Thermal ellipsoids are drawn for the asymmetric units with probability 30%). Dashed bonds represent the other parts of the 4-fold disordered 3-pyCN molecule. Symmetry codes: a = 1-*y*, 1-*x*, *z*; b = *y*, *x*, 1-*z*; c = 1-*x*, 1-*y*, 1-*z*; d = -*x*+*y*, *y*, 1.5-*z*.

(d)

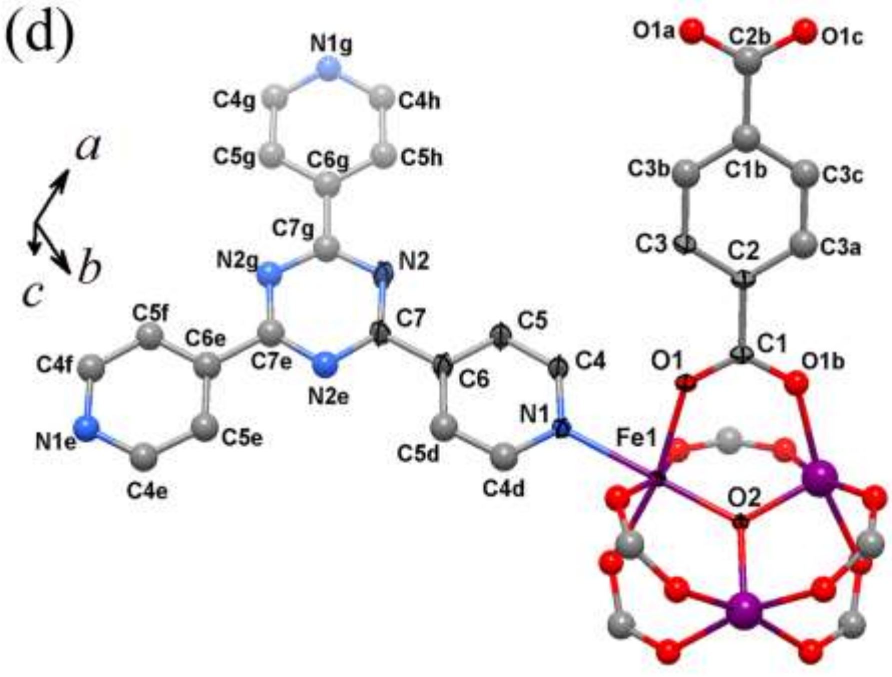

Supplementary Figure 1d. Perspective views of the coordination environments in MIL-88B-tpt. Hydrogen atoms are omitted for clarity (Thermal ellipsoids are drawn for the asymmetric units with probability 30%). Symmetry codes: a = 1-*y*, 1-*x*, *z*; b = *y*, *x*, 1-z; *c* = 1-*x*, 1-*y*, 1-*z*; d = -*x*+*y*, *y*, 1.5-*z*; e = -*y*, *x*-*y*, *z*; f = -*y*, -*x*, 1.5-z; g = -*x*+*y*, -*x*, *z*; h = *x*, *x*-*y*, 1.5-z.

(e)

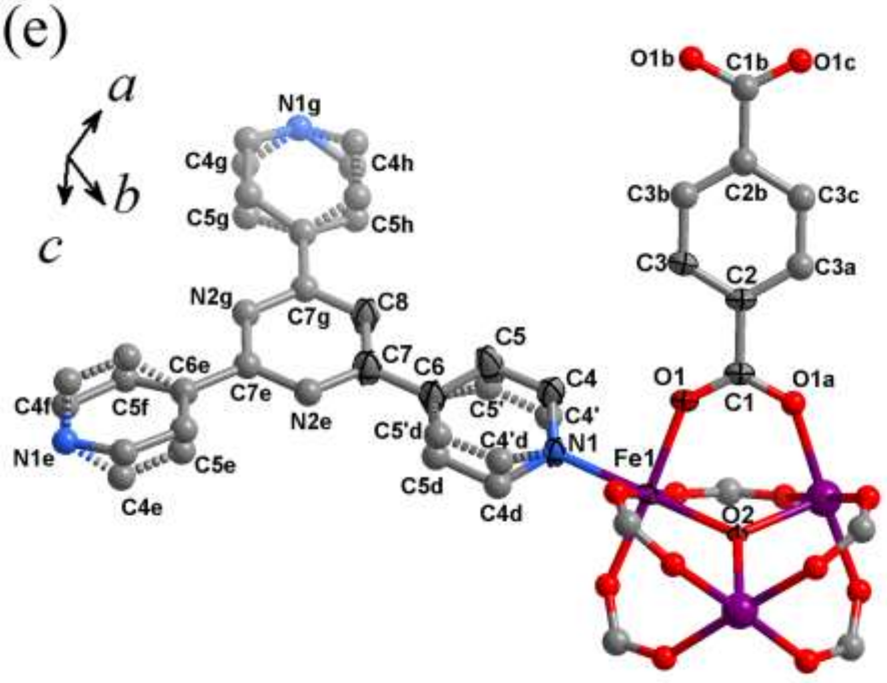

Supplementary Figure 1e. Perspective views of the coordination environments in MIL-88B-tpb. Hydrogen atoms are omitted for clarity (Thermal ellipsoids are drawn for the asymmetric units with probability 30%). Dashed bonds represent another part of the 2-fold disordered pyridyl group. Symmetry codes: a = 1-*y*, 1-*x*, *z*; b = *y*, *x*, 1-z; *c* = 1-*x*, 1-*y*, 1-z; d = -*x*+*y*, *y*, 1.5-*z*; e = -*y*, *x*-*y*, *z*; f = -*y*, -*x*, 1.5-*z*; g = -*x*+*y*, -*x*, *z*; h = *x*, *x*-*y*, 1.5-*z*.

(f)

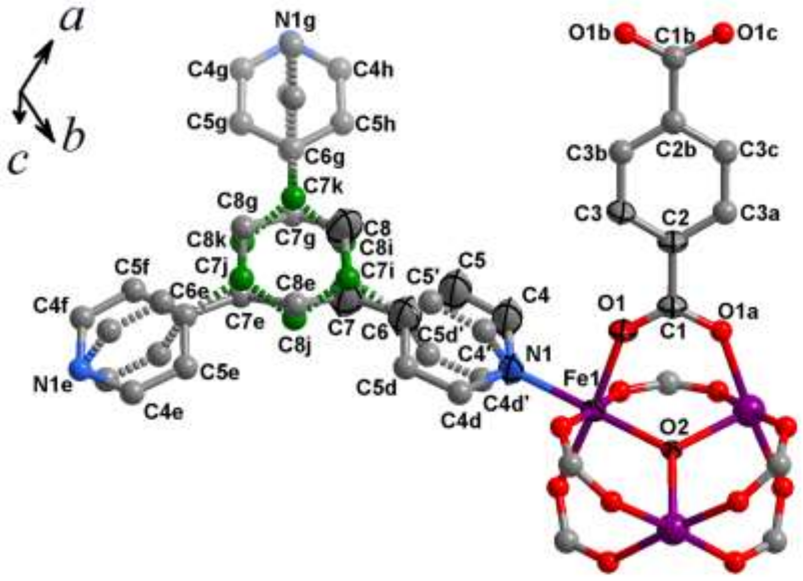

Supplementary Figure 1f. Perspective views of the coordination environments in MIL-88B-tpc. Hydrogen atoms are omitted for clarity (Thermal ellipsoids are drawn for the asymmetric units with probability 30%). Dashed bonds represent another part of the 2-fold disordered pyridyl/cyclohexane group. Symmetry codes: a = 1-*y*, 1-*x*, *z*; b = *y*, *x*, 1-z; *c* = 1-*x*, 1-*y*, 1-z; d = -*x*+*y*, *y*, 1.5-z; e = -*y*, *x*-*y*, *z*; f = -*y*, -*x*, 1.5-*z*; g = -*x*+*y*, -*x*, *z*; h = *x*, *x*-*y*, 1.5-*z*; i = -*x*+*y*, *y*, 1.5-*z*; j = -*y*, -*x*, 1.5-*z*; k = *x*, *x*-*y*, 1.5-*z*.

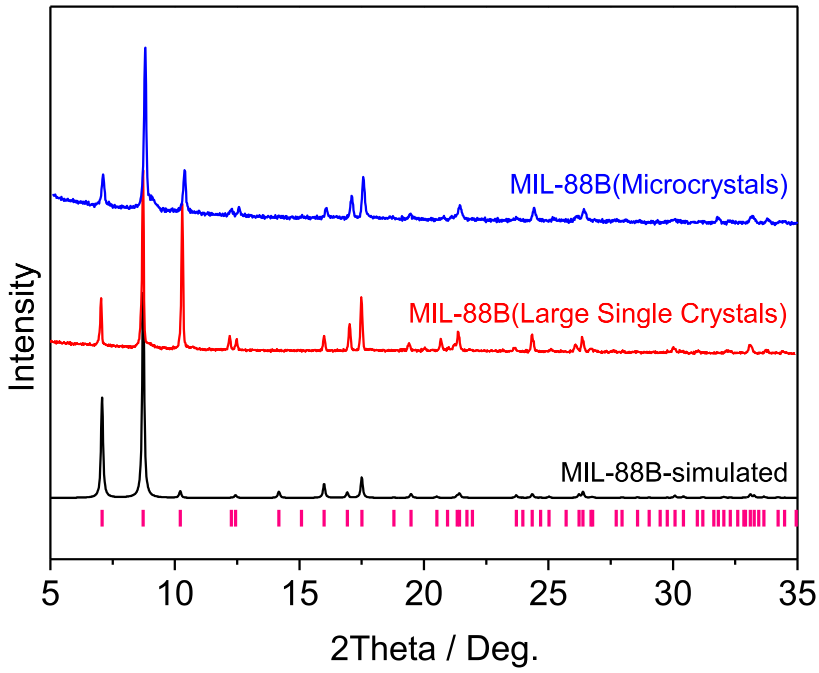

Supplementary Figure 2. PXRD patterns of MIL-88B.

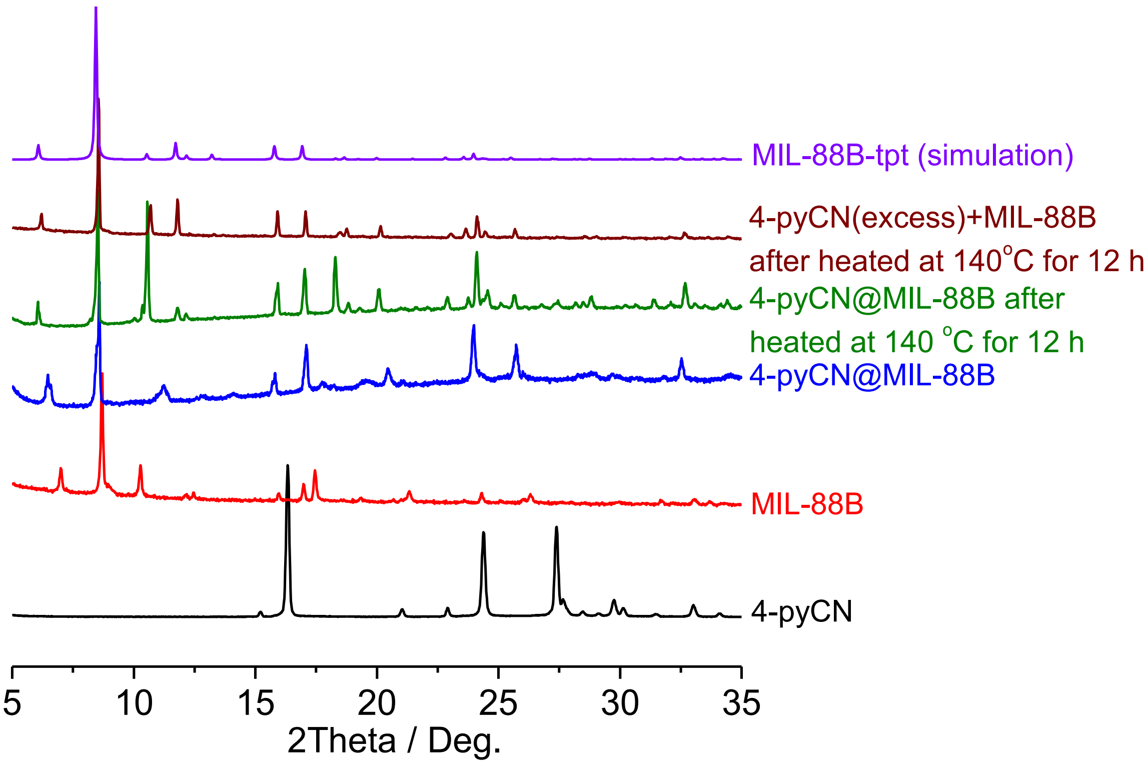

Supplementary Figure 3a. PXRD patterns for the CTC reactions using 4-pyCN as the monomers. Due to the loading of 4-pyCN in the flexible MIL-88B framework, 4-pyCN@MIL-88B possesses an expanded lattice compared with MIL-88B, but still much smaller than that of MIL-88B-tpt, indicating the loading of 4-pyCN into the flexible crystal and absence of CTC reaction. No characteristic PXRD peak of 4-pyCN can be observed for 4-pyCN@MIL-88B, confirming the absence of excess 4-pyCN. No tpt can be detected by IR (Figure S4a) or ESI-MS (Figure S5a) for 4-pyCN@MIL-88B, confirming that the CTC reaction has not started.

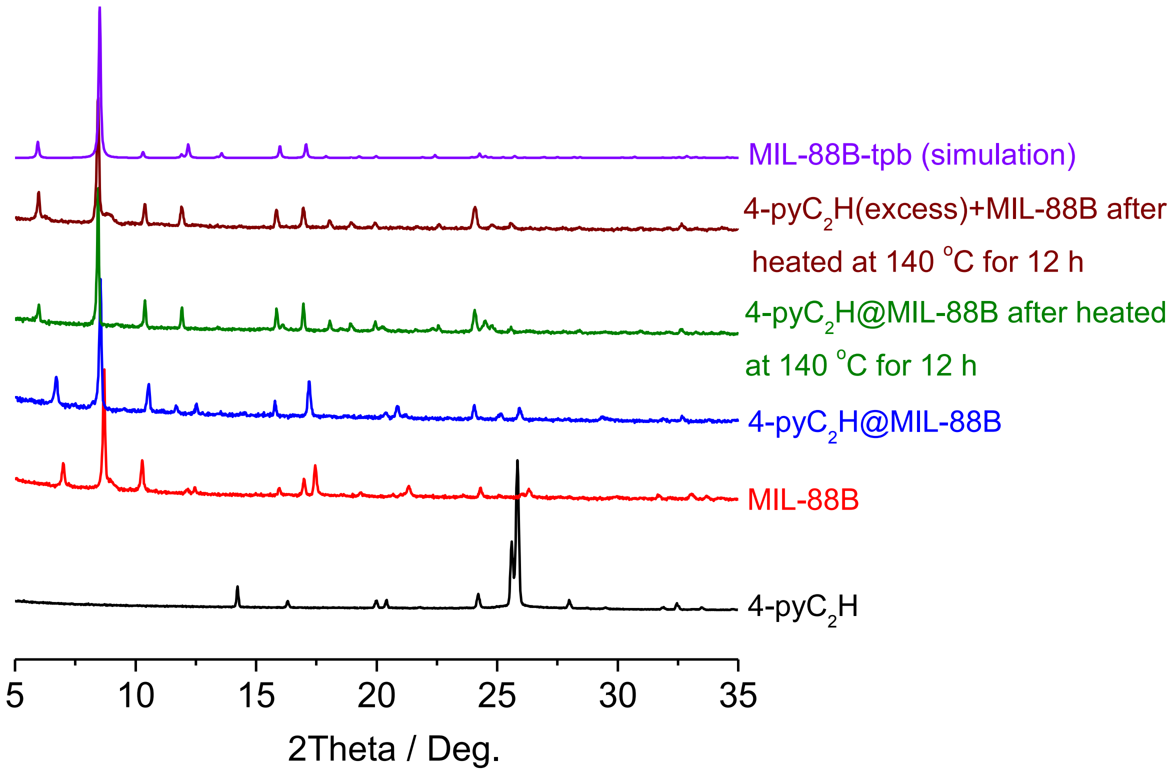

Supplementary Figure 3b. PXRD patterns for the CTC reaction using 4-pyC_2H as the monomers. Similar to 4-pyCN@MIL-88B, 4-pyC_2H@MIL-88B also possesses an expanded lattice compared with MIL-88B, but still much smaller than that of MIL-88B-tpb, indicating the loading of 4-pyC_2H into the flexible crystal and absence of CTC reaction. No characteristic PXRD peak of 4-pyC_2H can be observed for 4-pyC_2H@MIL-88B, confirming the absence of excess 4-pyC_2H. No tpb can be detected by IR (Figure S4b) or ESI-MS (Figure S5b) for 4-pyC_2H@MIL-88B, confirming that the CTC reaction has not started.

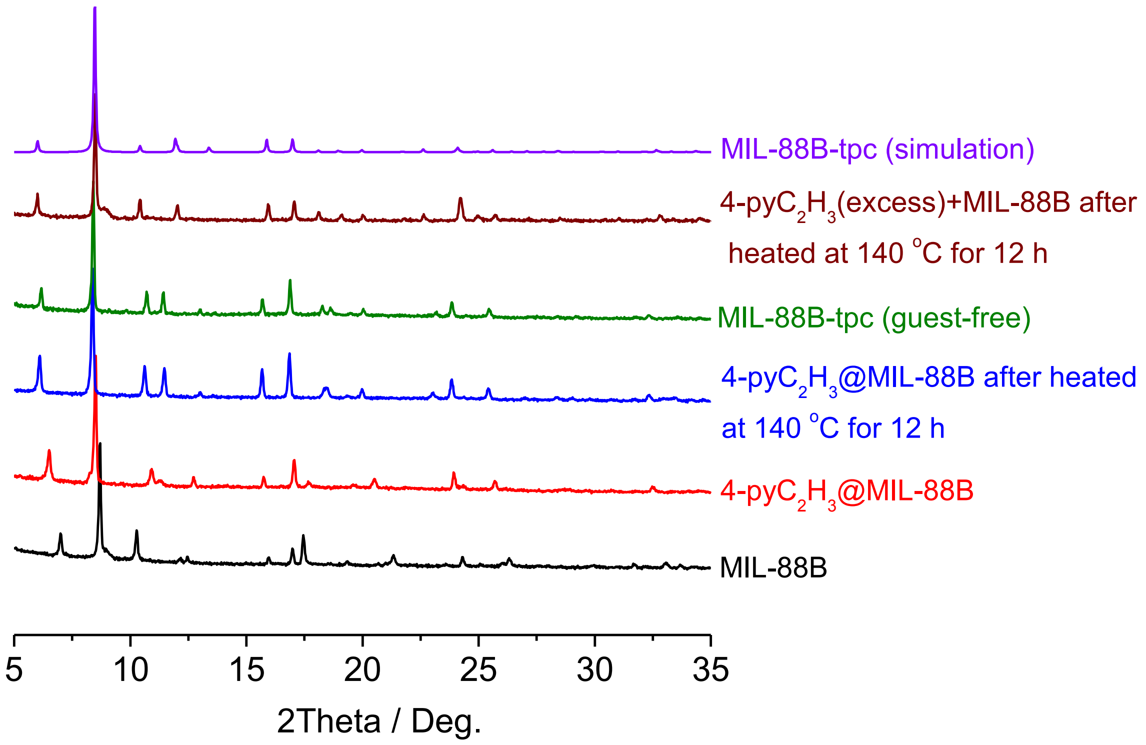

Supplementary Figure 3c. PXRD patterns for the CTC reaction using 4-pyC_2H_3 as the monomers. Similar to 4-pyCN@MIL-88B and 4-pyC_2H@MIL-88B, 4-pyC_2H_3@MIL-88B possesses an expanded lattice compared with MIL-88B, but still much smaller than that of MIL-88B-tpc, indicating the loading of 4-pyC_2H_3 into the flexible crystal and absence of CTC reaction. No tpc can be detected by ESI-MS for 4-pyC_2H_3@MIL-88B (Figure S5c), confirming that the CTC reaction has not started. The PXRD pattern of 4-pyC_2H_3(excess)+MIL-88B after heated at 140 °C for 12 h matches with that simulated from the single-crystal structure of MIL-88B-tpc. After removal of guest, guest-free MIL-88B-tpc with a shrunk lattice was obtained, which can be attributed to the flexibility of the nonplanar cyclohexane core of tpc. The PXRD pattern of 4-pyC_2H_3@MIL-88B after heated at 140 °C for 12 h matches with that of guest-free MIL-88B-tpc instead of that simulated from the single-crystal structure of MIL-88B-tpc, because the CTC reaction without excess 4-pyC_2H_3 is solvent-free.

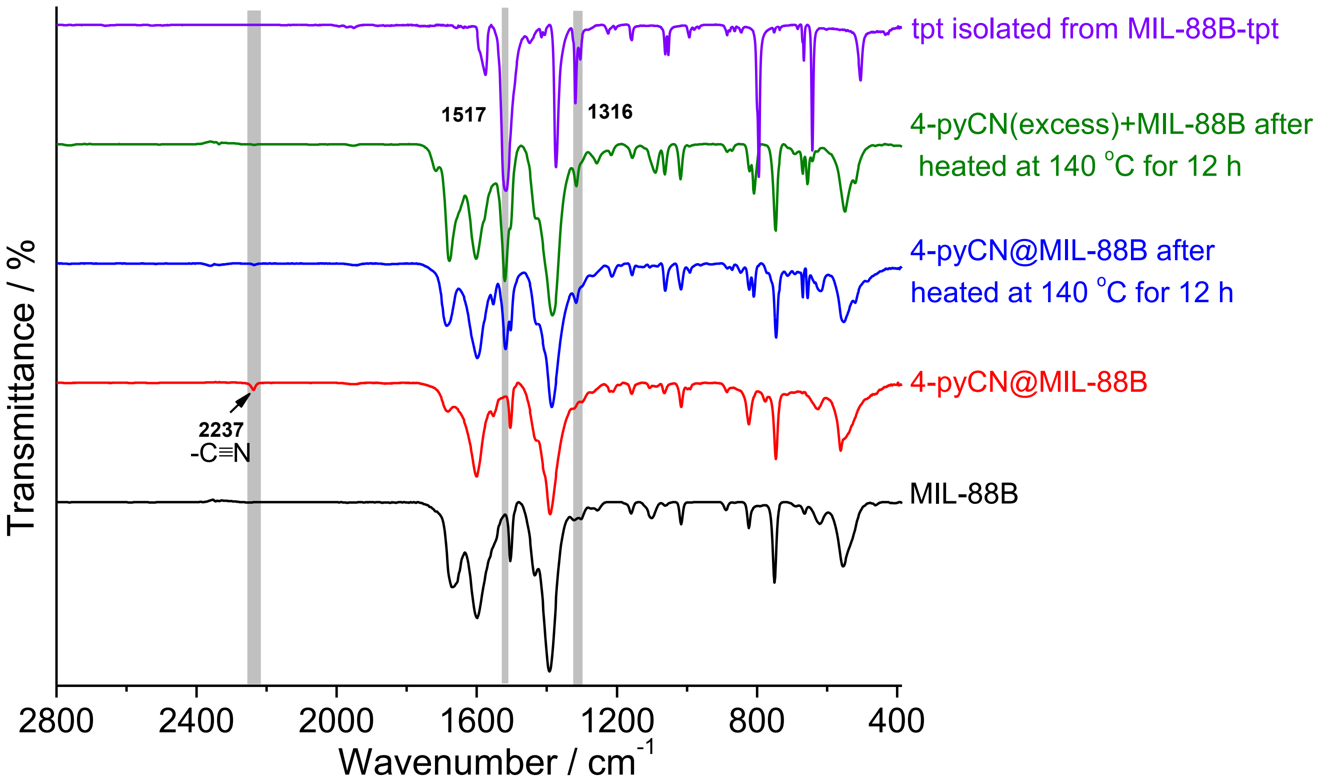

Supplementary Figure 4a. IR spectra for the CTC reactions using 4-pyCN as the monomers. The characteristic carbonitrile stretching band of the 4-pyCN at 2237 cm^{-1} and aromatic C-N stretching band characteristic of the triazine units at 1517 and 1316 cm^{-1} are highlighted.

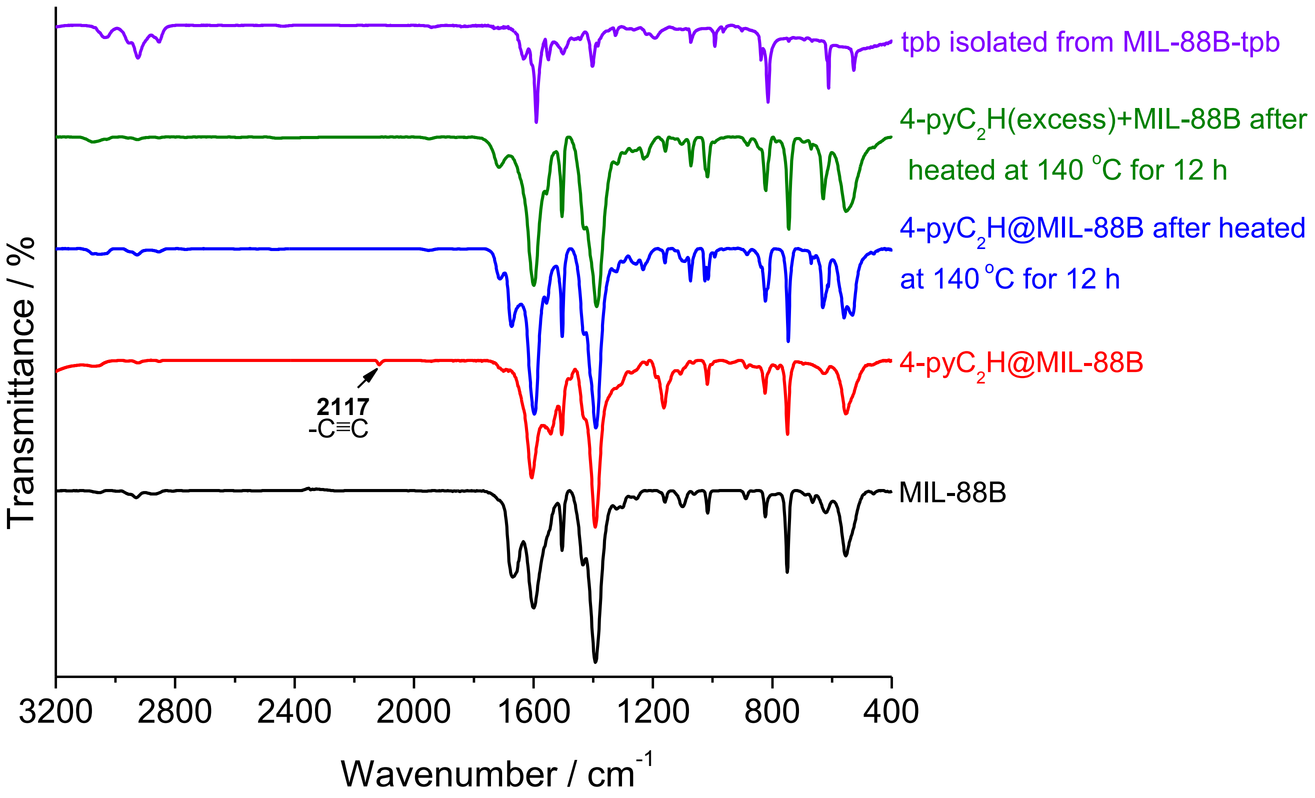

Supplementary Figure 4b. IR spectra for the CTC reactions using 4-pyC_2H as the monomers. The characteristic stretching band of the ethynyl of 4-pyC_2H at 2117 cm^{-1} is highlighted.

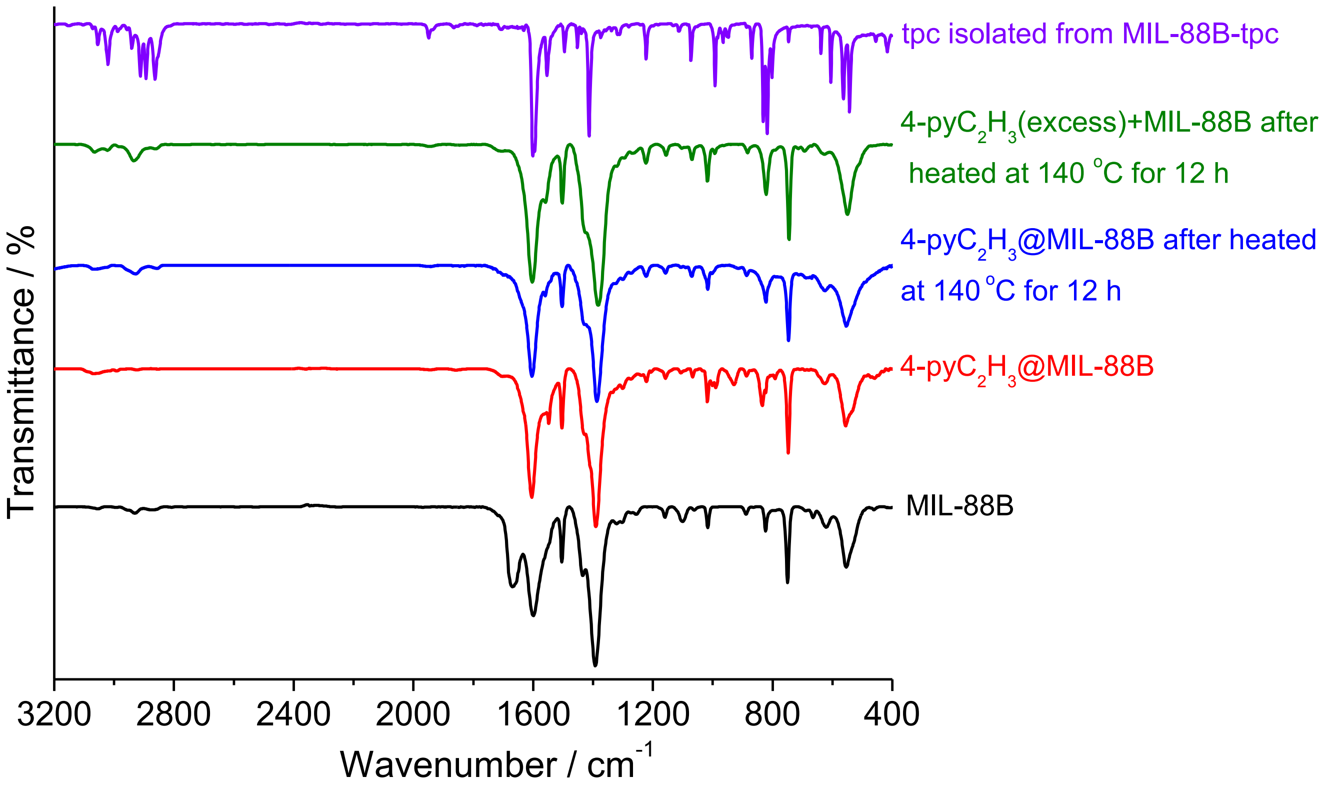

Supplementary Figure 4c. IR spectra for the CTC reactions using 4-pyC_2H_3 as the monomers.

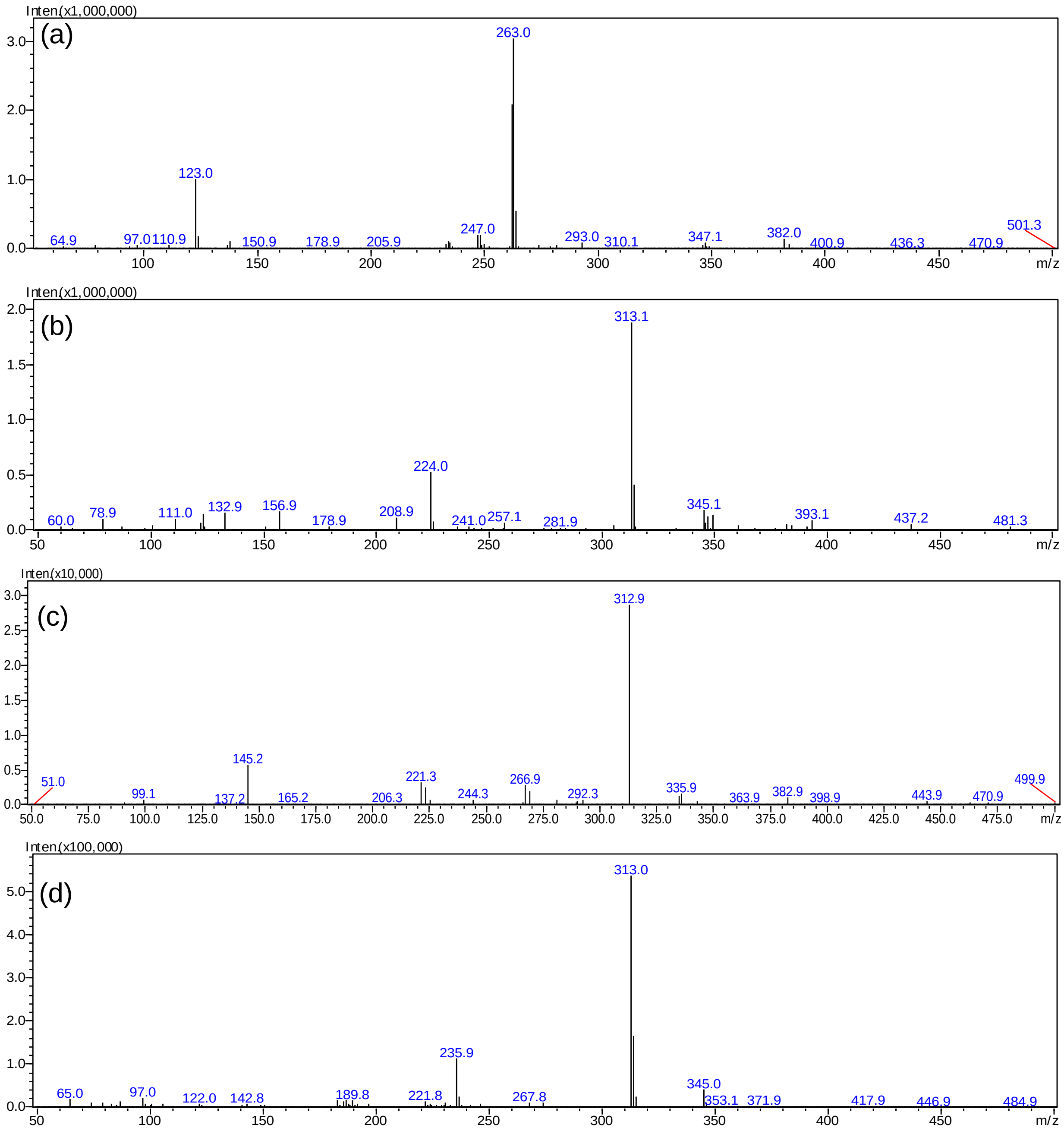

Supplementary Figure 5a. ESI(+)-MS for the CTC reactions using 4-pyCN as the monomers. (a) acid-digested 4-pyCN@MIL-88B (the hydrolysis product of 4-pyCN, 4-pyridinecarboxamide can be detected; no tpt can be detected); (b) acid-digested 4-pyCN@MIL-88B after heated at 140 °C for 12 h (tpt can be detected); (c) acid-digested 4-pyCN(excess)+MIL-88B after heated at 140 °C for 12 h (tpt can be detected); (d) tpt isolated from MIL-88B-tpt. Calculated *M*/*z* for protonated 4-pyridinecarboxamide and tpt are 123 and 313, respectively.

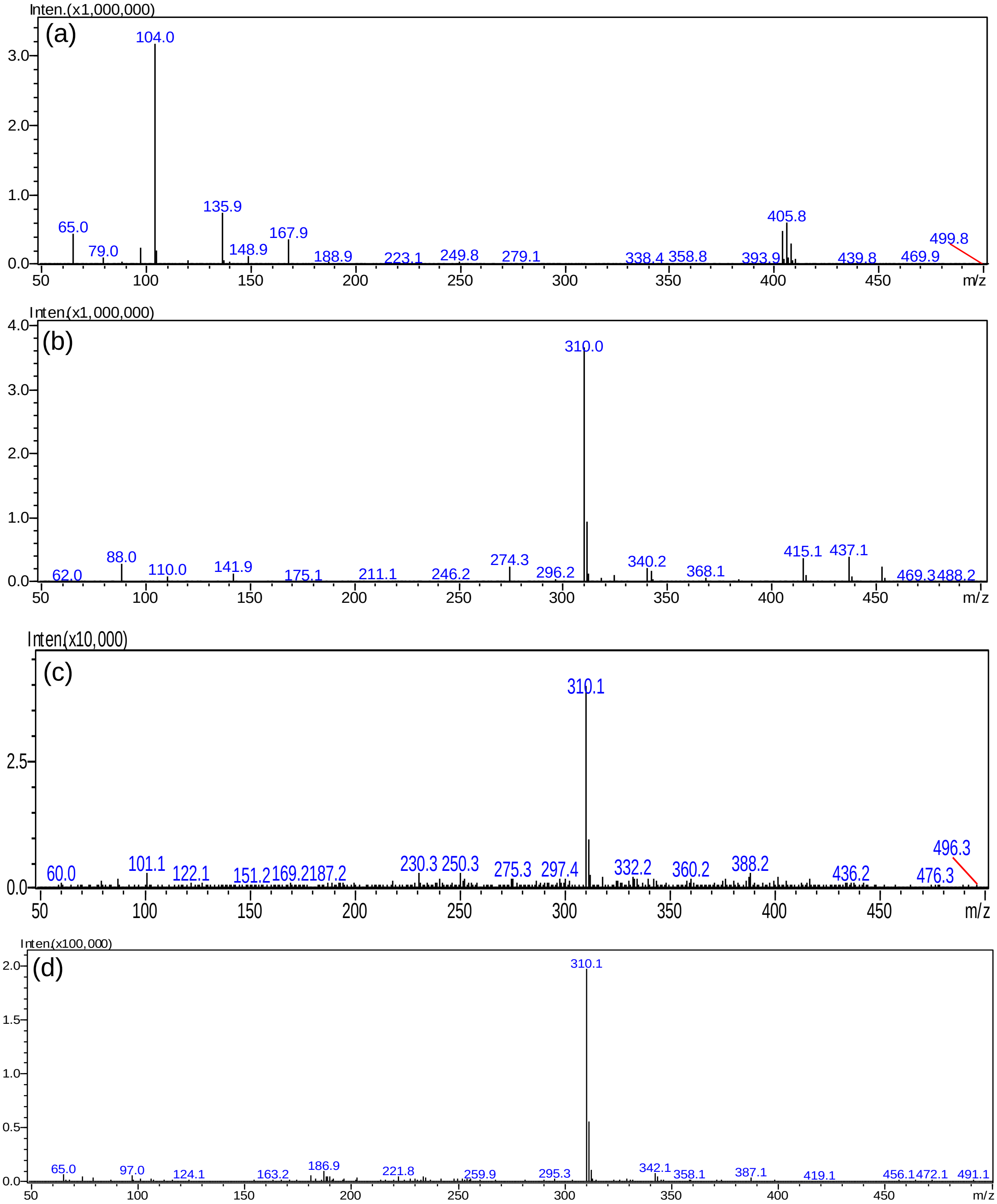

Supplementary Figure 5b. ESI(+)-MS for the CTC reactions using 4-pyC_2H as the monomers. (a) acid-digested 4-pyC_2H@MIL-88B [4-pyC_2H was detected, no tpb can be detected]; (b) acid-digested 4-pyC_2H@MIL-88B after heated at 140 ^oC for 12 h (tpb can be detected); (c) acid-digested 4-pyC_2H(excess)+MIL-88B after heated at 140 ^oC for 12 h (tpb can be detected); (d) tpb isolated from MIL-88B-tpb. Calculated *M*/*z* for protonated 4-pyC_2H and tpb are 104 and 310, respectively.

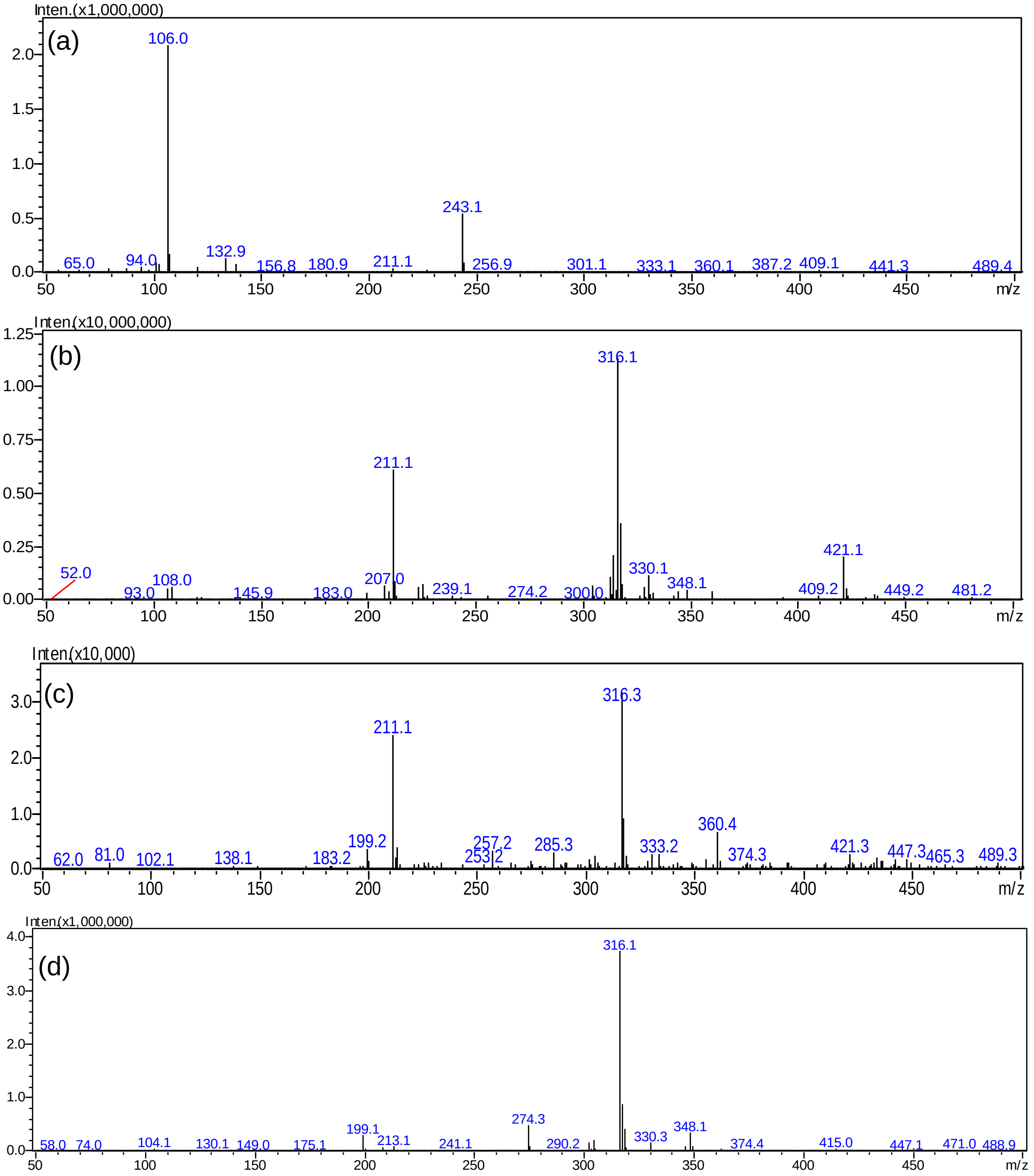

Supplementary Figure 5c. ESI(+)-MS for the CTC reactions using 4-pyC_2H_3 as the monomers. (a) acid-digested 4-pyC_2H_3@MIL-88B (4-pyC_2H_3 was detected, no tpc can be detected); (b) acid-digested 4-pyC_2H_3@MIL-88B after heated at 140 ^oC for 12 h (tpc can be detected); (c) acid-digested 4-pyC_2H_3(excess)+MIL-88B after heated at 140 ^oC for 12 h (tpc can be detected); (d) tpc isolated from MIL-88B-tpc. Calculated *M*/*z* for protonated 4-pyC_2H_3 and tpc are 106 and 316, respectively.

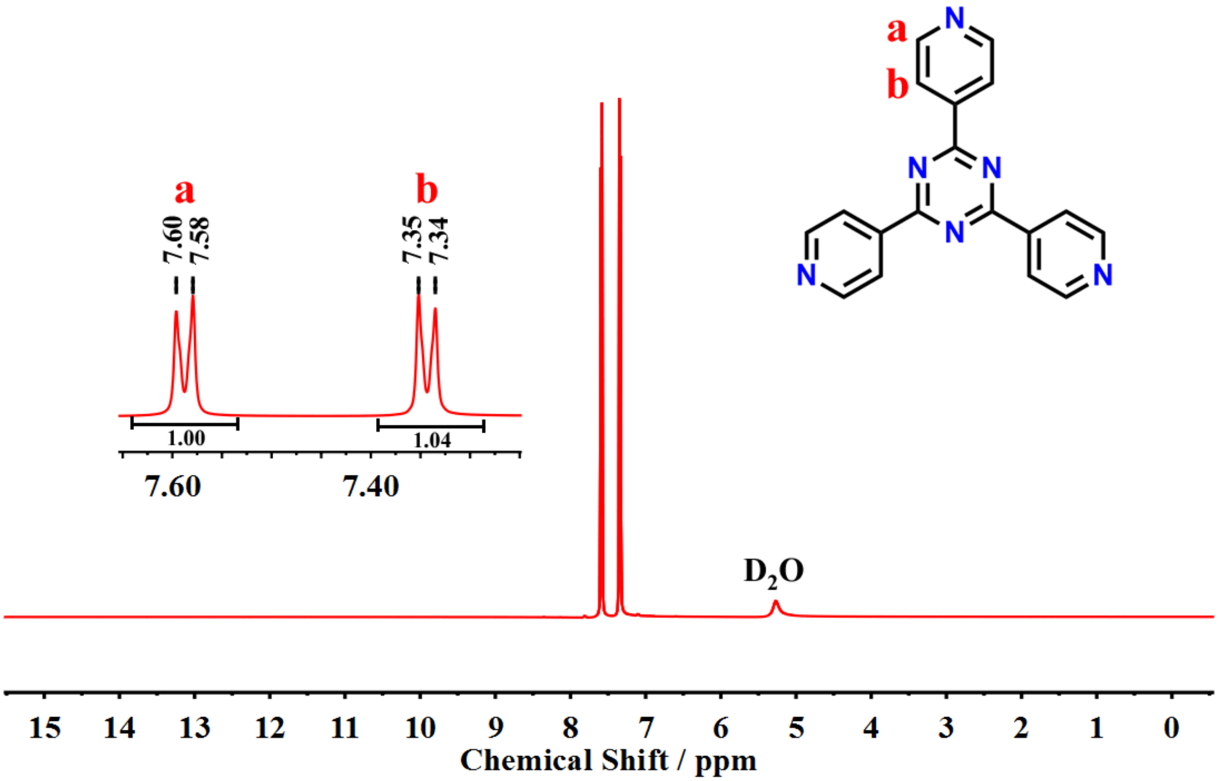

Supplementary Figure 6. ^{1}H NMR (400 MHz, DCl, 298 K) spectra of tpt isolated from MIL-88B-tpt.

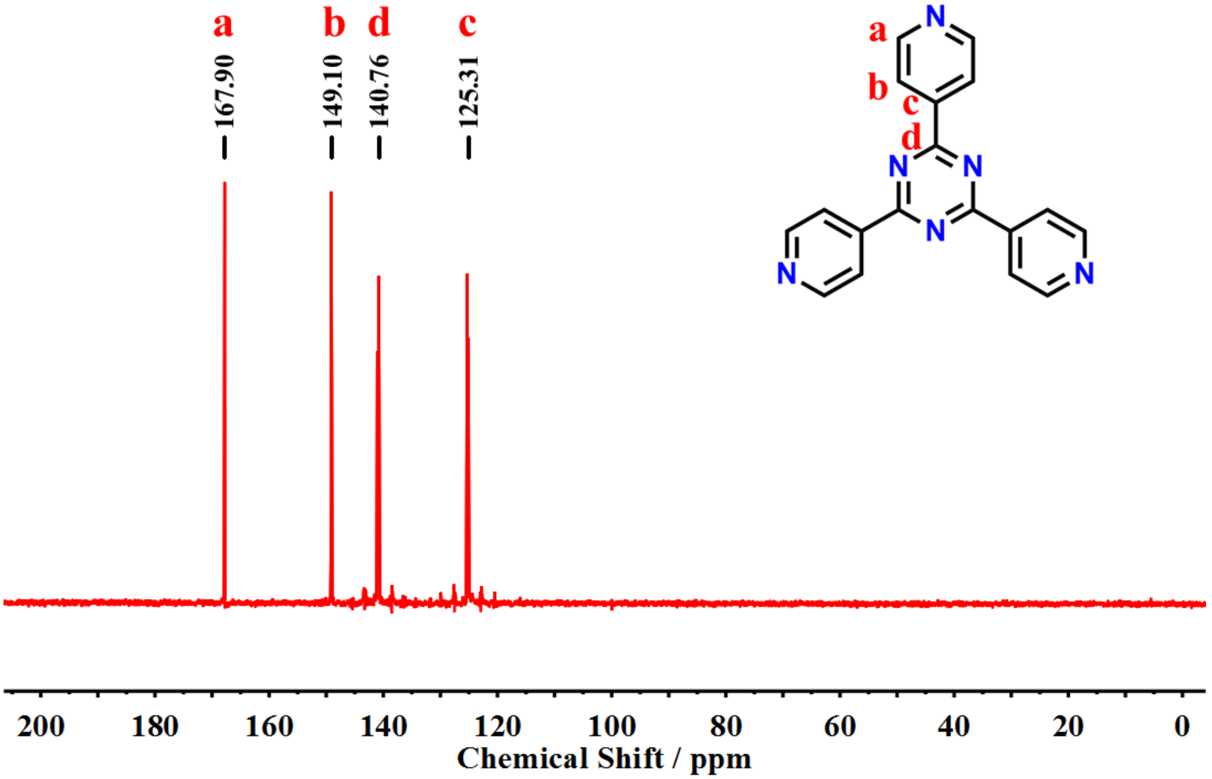

Supplementary Figure 7. ^{13}C NMR (400 MHz, DCl, 298 K) spectrum of tpt isolated from MIL-88B-tpt.

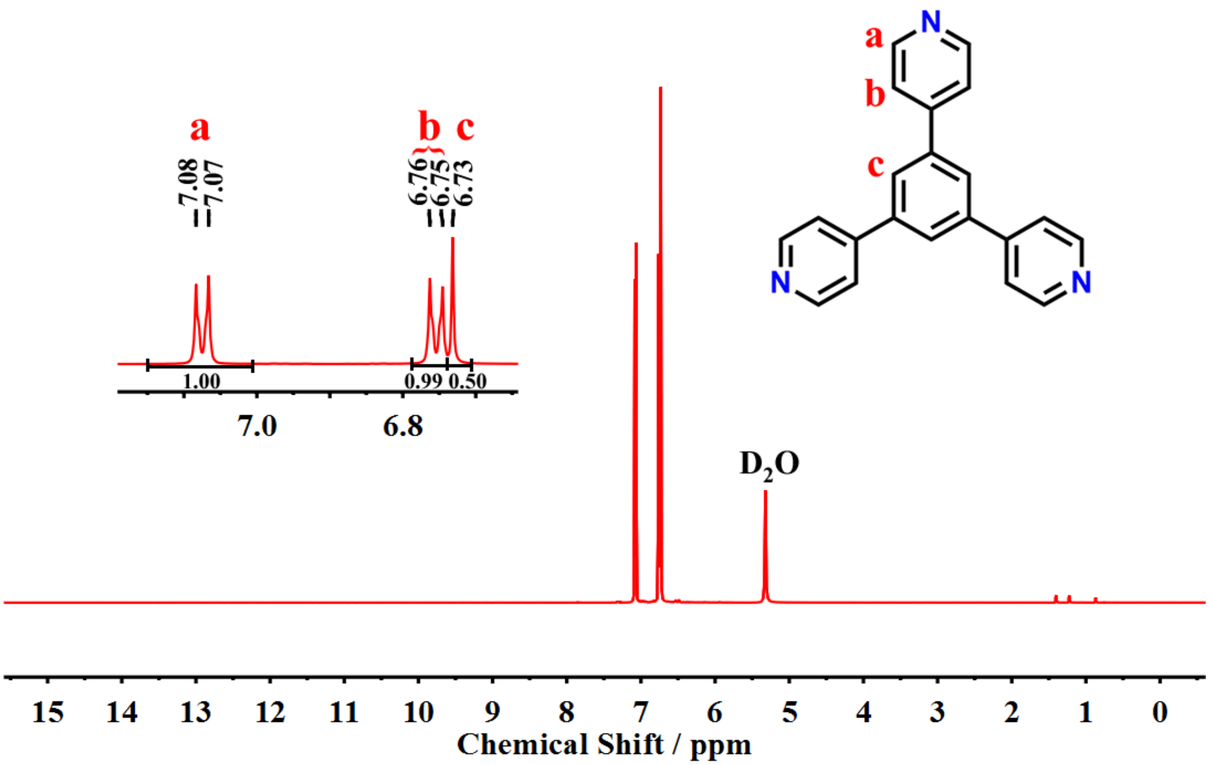

Supplementary Figure 8. ^{1}H NMR (400 MHz, DCl, 298 K) spectra of tpb isolated from MIL-88B-tpb.

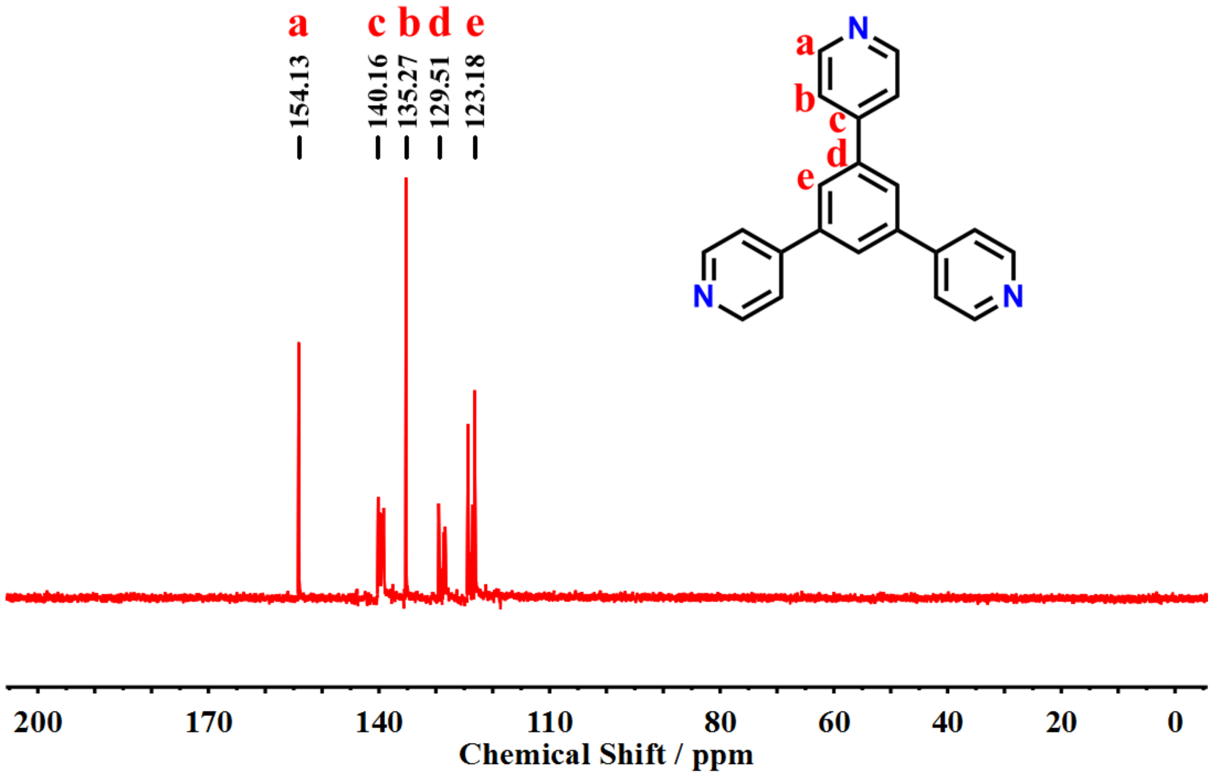

Supplementary Figure 9. ^{13}C NMR (400 MHz, DCl, 298 K) spectrum of tpb isolated from MIL-88B-tpb.

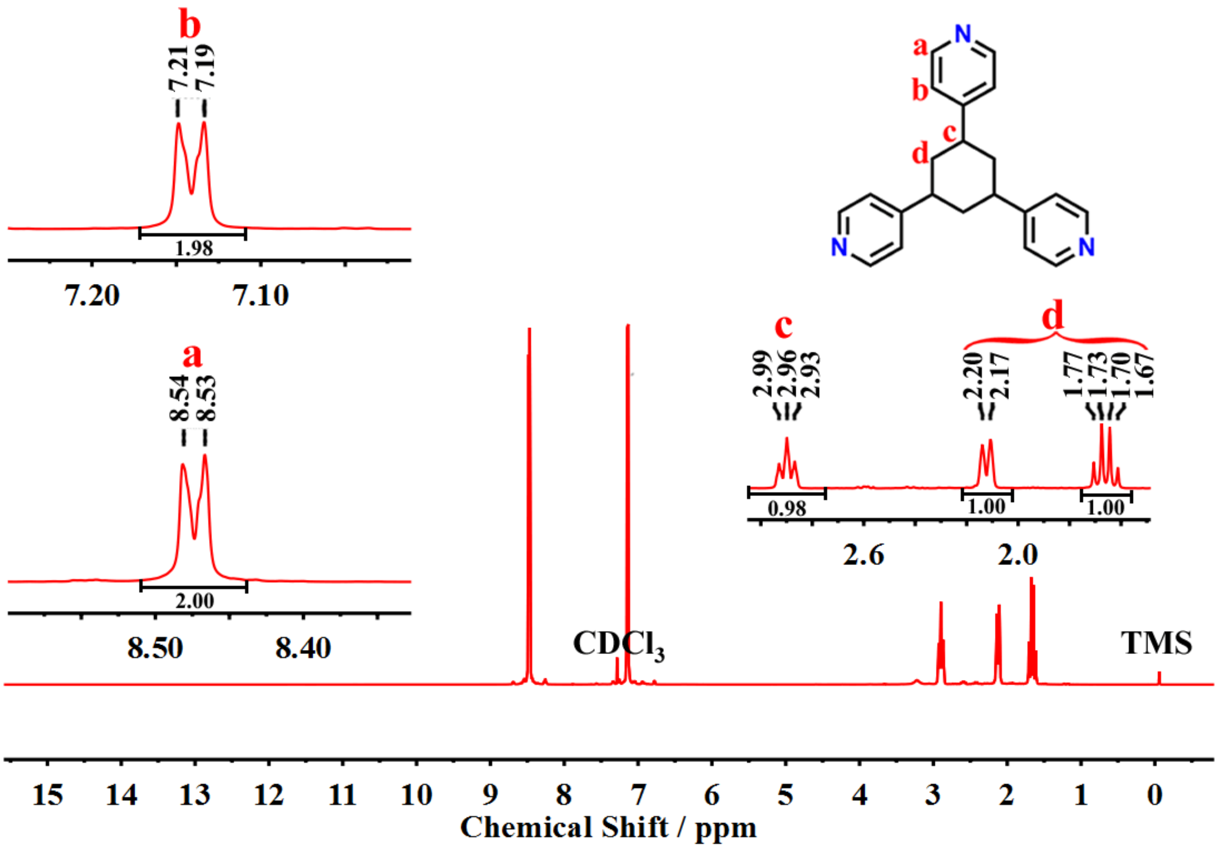

Supplementary Figure 10. ^{1}H NMR (400 MHz, $CDCl_3$, 298 K) spectra of tpc isolated from MIL-88B-tpc. There are two types of H atoms on the methylene moieties of the cyclohexane ring (see ref *J. Am. Chem. Soc.* **2008**, *130*, 9566).

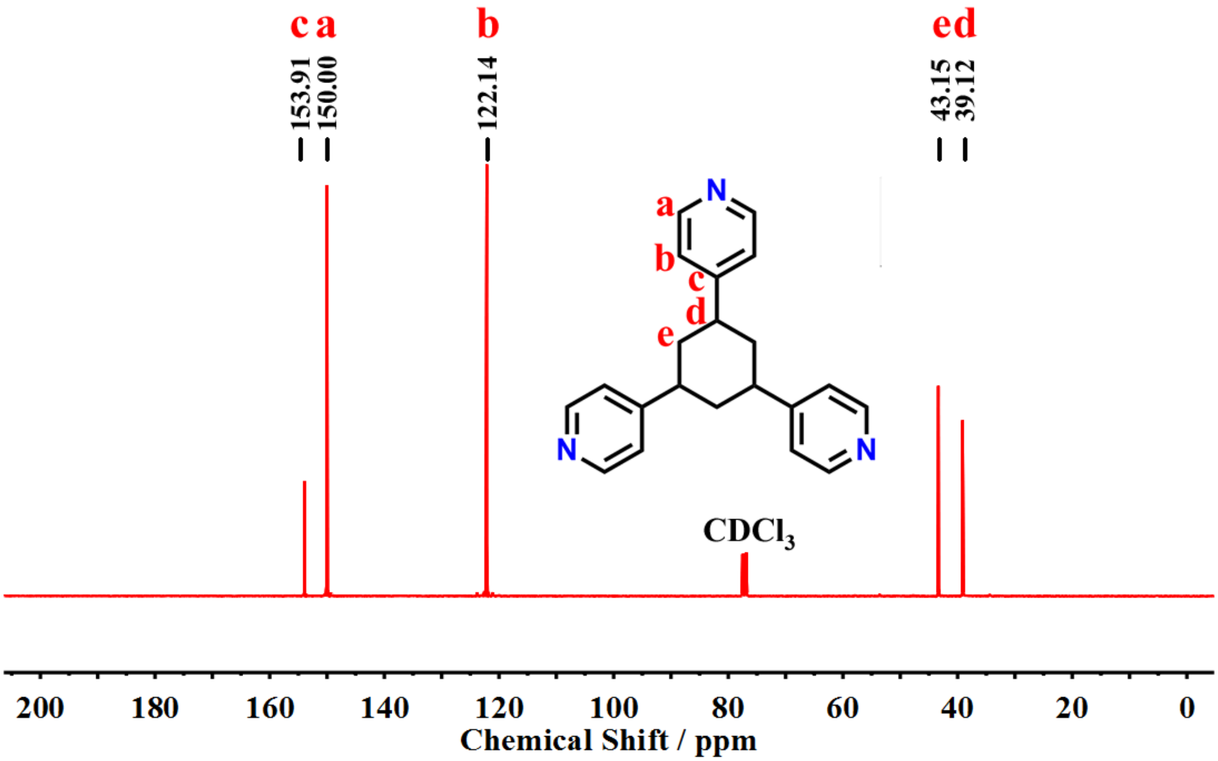

Supplementary Figure 11. ^{13}C NMR (400 MHz, $CDCl_3$, 298 K) spectrum of tpc isolated from MIL-88B-tpc.

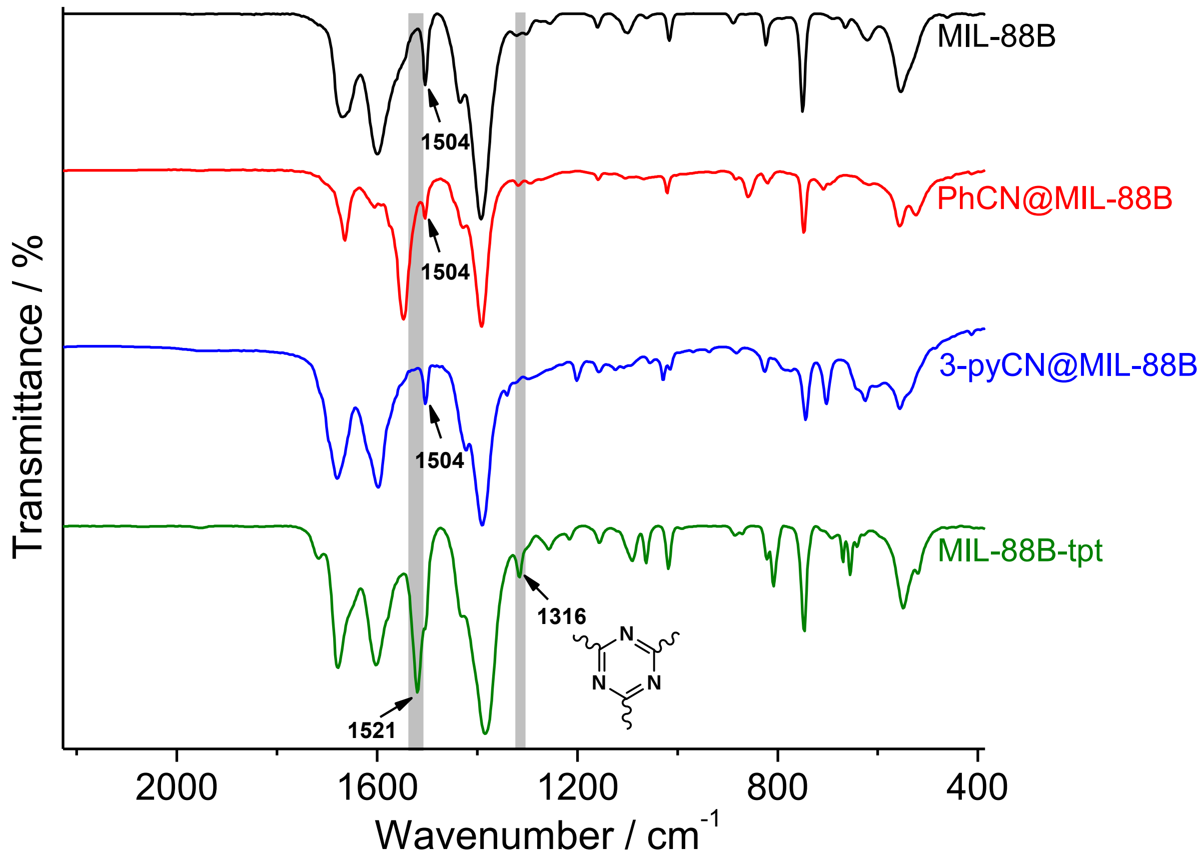

Supplementary Figure 12. IR spectra of MIL-88B&3-pyCN and MIL-88B&PhCN heated at 180 °C for 24 h. The residual 3-pyCN/PhCN in the samples was removed by sublimation and the trimeric product of 3-pyCN/PhCN was not detected at characteristic absorption bands at 1521 or 1316 cm^{-1}.

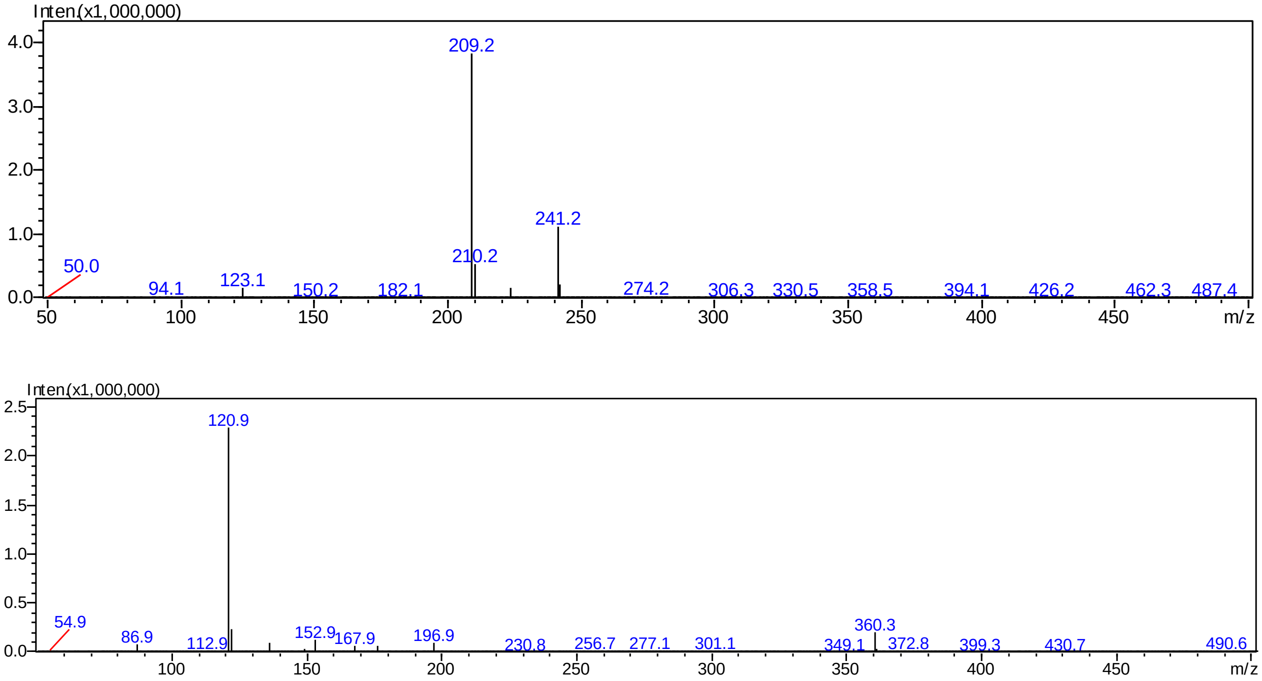

Supplementary Figure 13. ESI(+)-MS of acid-digested MIL-88B&3-pyCN (up) and MIL-88B&PhCN (bottom) heated at 180 ^{o}C for 24 h. The residual 3-pyCN/PhCN in the samples was removed by sublimation and the trimeric product of 3-pyCN/PhCN was not detected at M/z = 313.1 or 310.1 for protonated 2,4,6-tri(pyridin-3-yl)-1,3,5-triazine and 2,4,6-triphenyl-1,3,5-triazine.

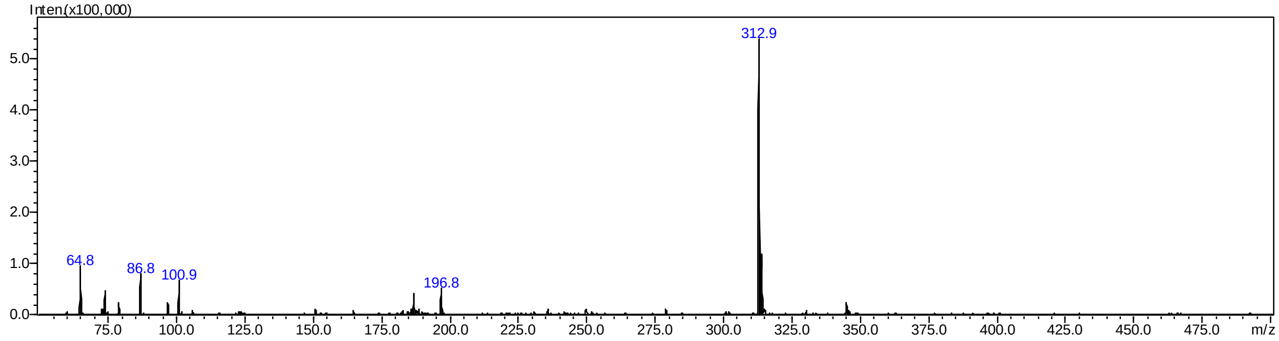

Supplementary Figure 14. ESI(+)-MS of acid-digested MIL-88B&(4-pyCN+PhCN) after heated at 180 °C for 24 hours. The reactant amounts were 0.10 g for MIL-88B, 1.50 g for 4-pyCN and 1.50 g for PhCN. The residual 4-pyCN and PhCN in the samples was removed by sublimation. Only $Htpt^+$ can be detected (at M/z = 313), while the protonated trimeric product of neither one 4-pyCN plus two PhCN, two 4-pyCN plus one PhCN, nor three PhCN was detected at M/z = 311, 312, and 310, respectively.

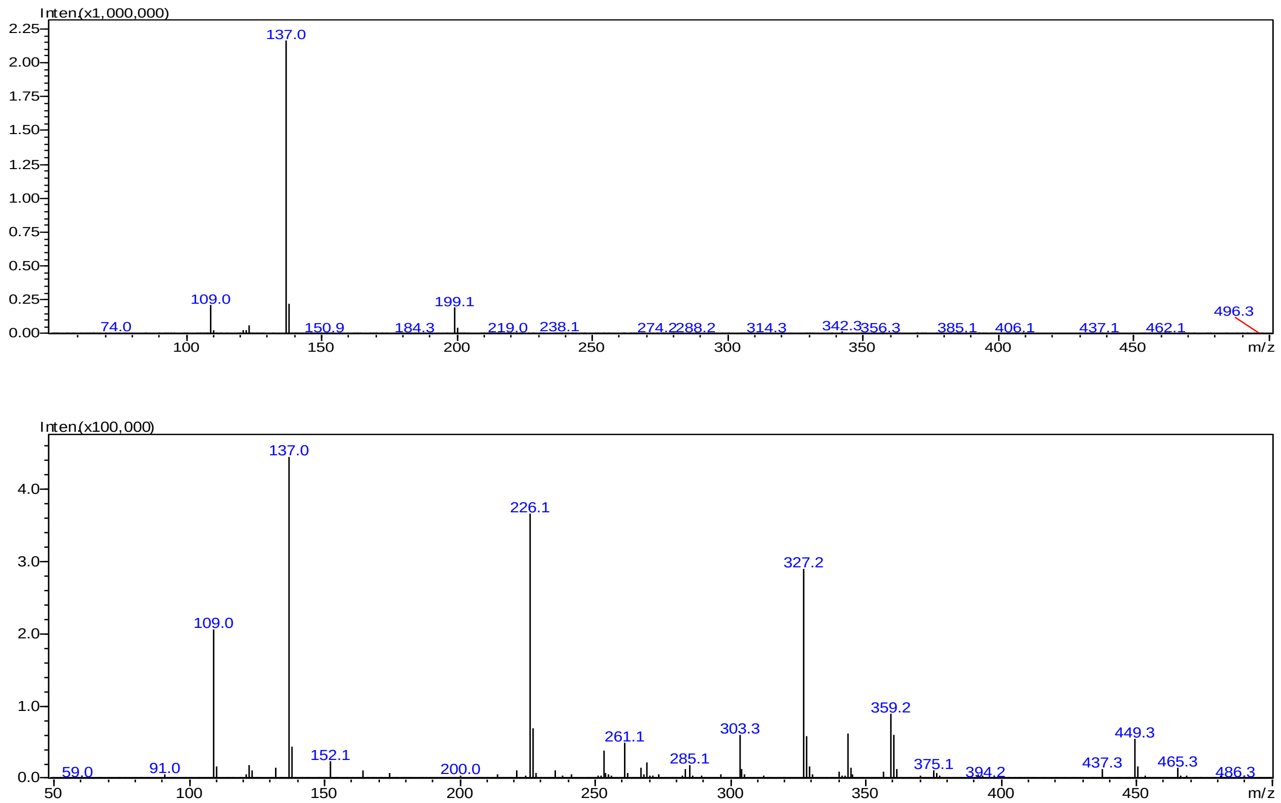

Supplementary Figure 15. ESI(+)-MS analyses of tpt outside the Fe_3 triangular templating sites. A mixture of MIL-88B (100 mg) and 4-pyCN (3.00 g), after heated at 180 ^{o}C for 1 d, was washed sequentially by MeOH (10×3.0 mL) (top) and benzyl alcohol (5×3.0 mL) (bottom), which were concentrated and then analyzed by ESI(+)-MS. No $Htpt^+$ was detected at *M*/*z* = 313. Since the reaction time is much longer than that (4 h) necessary for completion of the CTC reaction, if the cyclotrimerization is not just occurred at the proposed triangle sites, tpt will accumulate and present not only at the Fe_3 trianglular templating site, but also in other places of the crystal (not coordinated with Fe(III)) and outside the crystal, which can be extracted by methanol and benzyl alcohol.

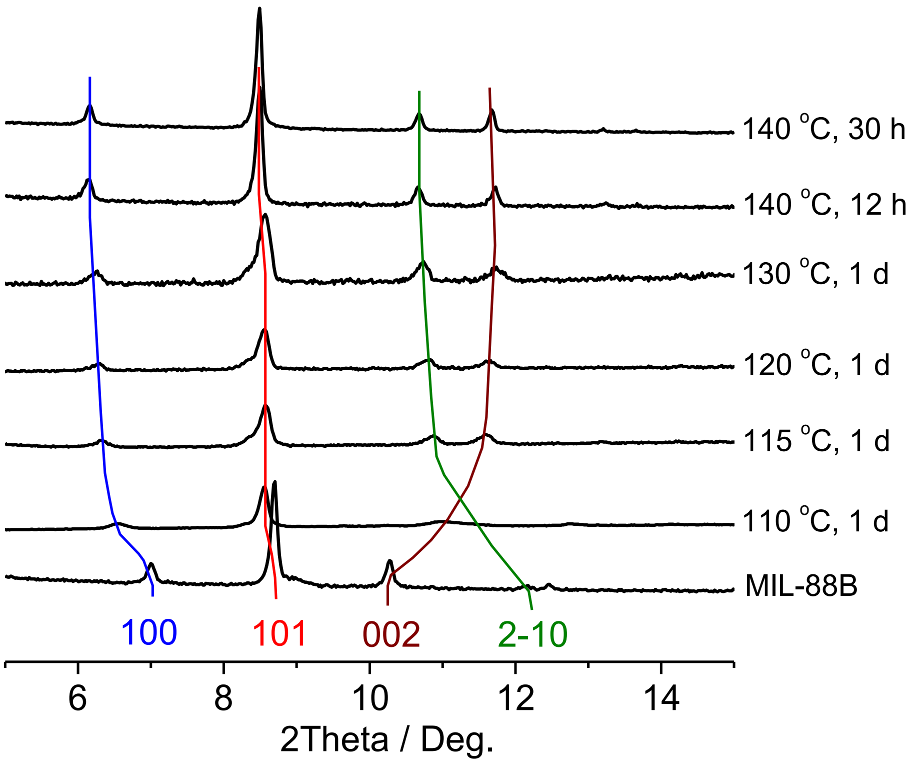

Supplementary Figure 16. Room-temperature PXRD patterns of MIL-88B&4-pyCN after heated at different temperatures and time. The residual 4-pyCN in the samples was washed off by DMF before measurement.

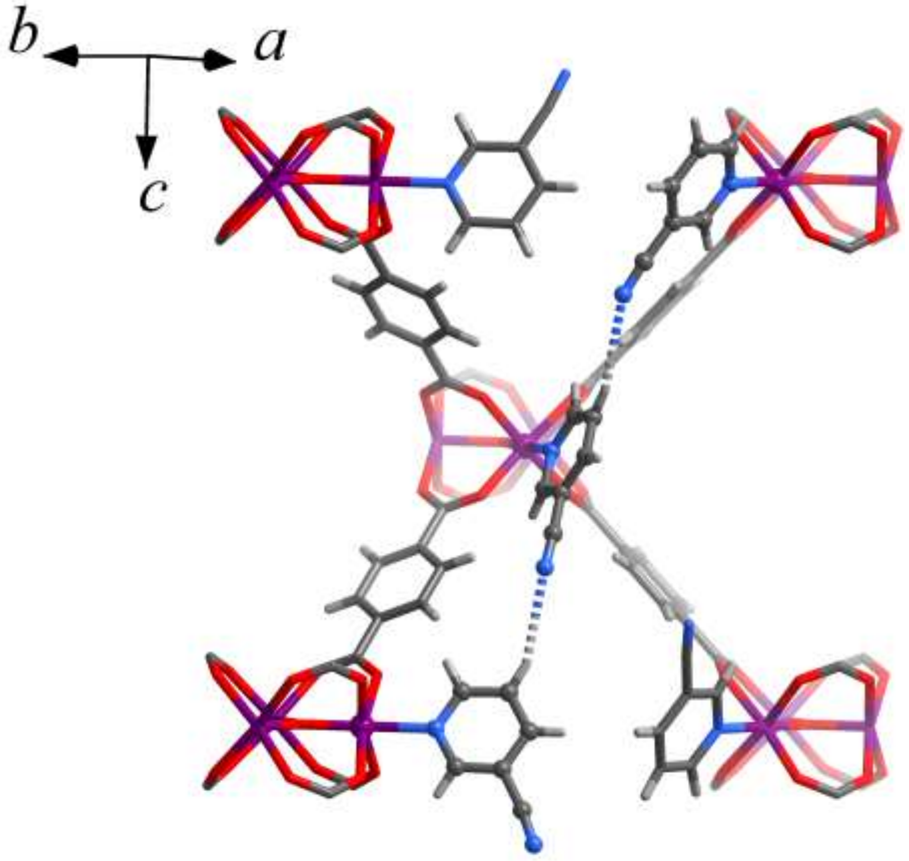

Supplementary Figure 17. Local structure around a 3-pyCN molecule in 3-pyCN@MIL-88B, showing the weak intermolecular C≡N···H−C hydrogen bonding interactions among the 3-pyCN molecules.

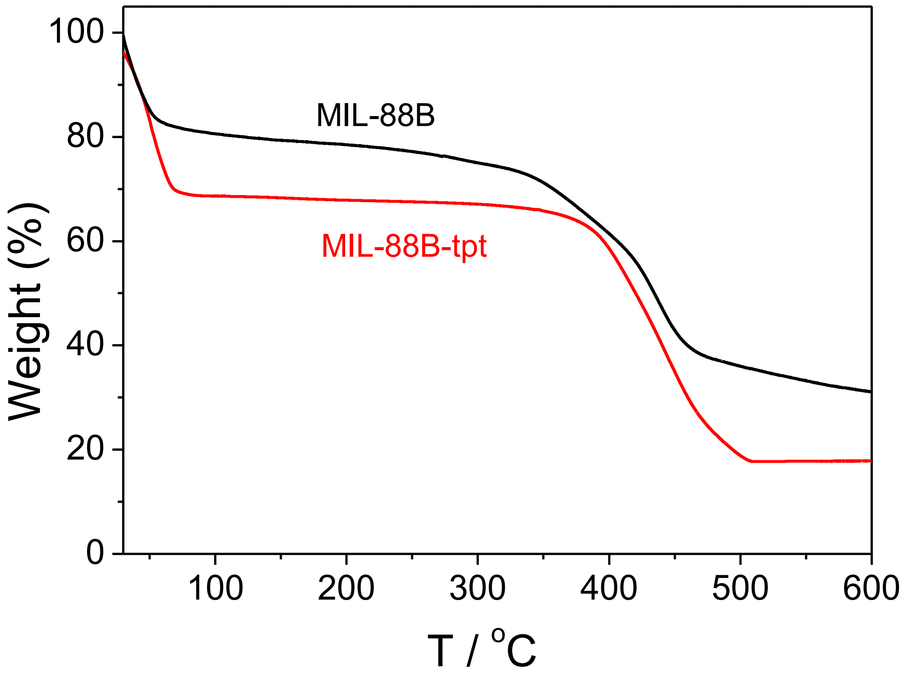

Supplementary Figure 18. TG curves of MIL-88B and MIL-88B-tpt. The samples used here were completely washed by hot MeOH using a Soxhlet extractor.

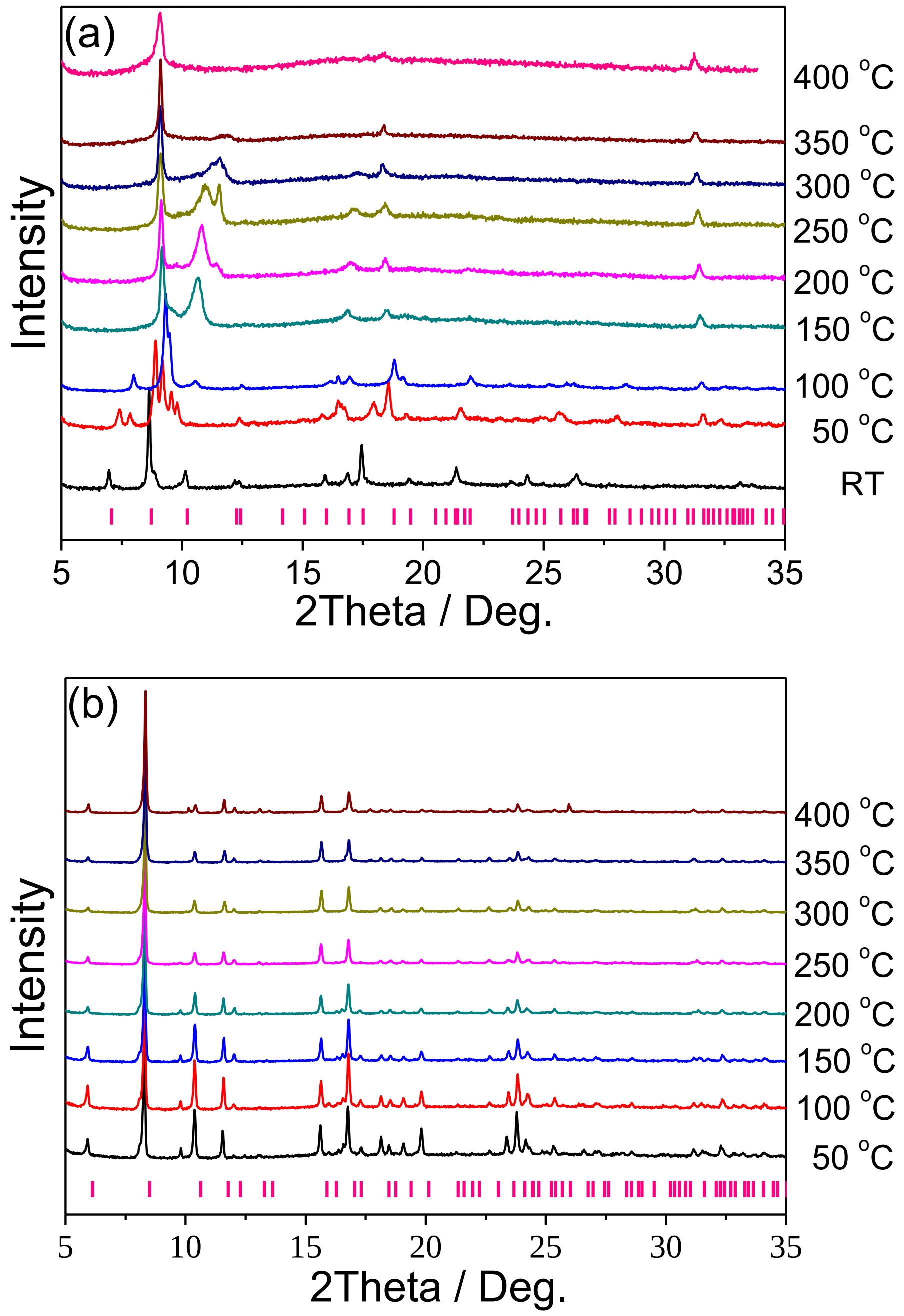

Supplementary Figure 19. PXRD patterns of (a) MIL-88B and (b) MIL-88B-tpt after heated at different temperatures under N_2 for 30 min.

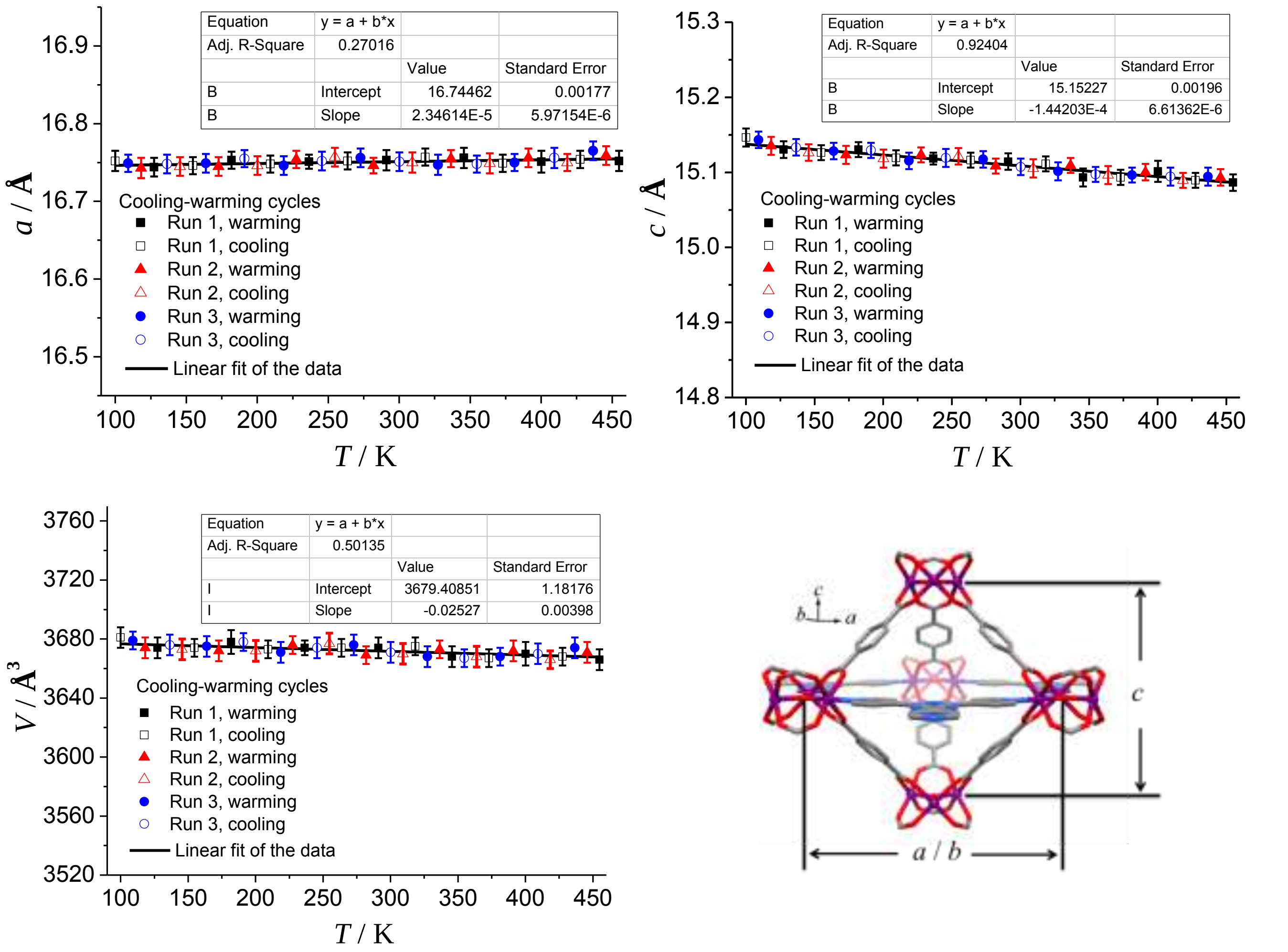

Supplementary Figure 20. Temperature- and guest-dependent cell parameters of MIL-88B-tpt. Obviously, the unit-cell parameters of MIL-88B-tpt were nearly unaffected by guest and temperature, giving zero thermal expansion across the *ab*-plane and small negative thermal expansion along the *c*-axis. Error bars show one estimated standard deviation. A representation of the axial length is depicted in the insert.

Supplementary Table 1. Crystallographic Data and Structural Refinements.

Complex	MIL-88B	MIL-88B-tpb	MIL-88B-tpc
Formula	$C_{24}H_{14}O_{15}FFe_3$	$C_{45}H_{27}O_{13}N_3Fe_3$	$C_{45}H_{33}O_{13}N_3Fe_3$
Formula weight	728.90	985.25	991.29
Temperature (K)	298(2)	298(2)	298(2)
Crystal system	Hexagonal	Hexagonal	Hexagonal
Space group	$P6_3/mmc$	$P6_3/mmc$	$P6_3/mmc$
a/Å	14.4162(3)	17.144(5)	16.9645(2)
c/Å	17.3040(3)	14.514(6)	14.8081(2)
V/Å^3	3114.43(14)	3694(3)	3690.73(10)
Z	2	2	2
ρ_{calc} (g cm^{-3})	0.779	0.886	0.892
μ (mm^{-1})	5.855	5.010	5.015
$R_1{}^a$ (I > 2σ)	0.0754	0.0661	0.0698
$wR_2{}^b$ (all data)	0.2380	0.1654	0.2140
S	1.051	1.062	1.048

$^aR_1 = \Sigma||F_o| - |F_c||/\Sigma|F_o|$. $^bwR_2 = [\Sigma w(F_o{}^2 - F_c{}^2)^2/\Sigma w(F_o{}^2)^2]^{1/2}$.

Supplementary Table 1. (Continued)

Complex	MIL-88B-tpt[c]	MIL-88B-tpt[d]	MIL-88B-tpt[e]
Formula	$C_{42}H_{24}O_{13}N_6Fe_3$	$C_{42}H_{24}O_{13}N_6Fe_3$	$C_{42}H_{24}O_{13}N_6Fe_3$
Formula weight	988.22	988.22	988.22
Temperature (K)	298(2)	361(2)	298(2)
Crystal system	Hexagonal	Hexagonal	Hexagonal
Space group	$P6_3/mmc$	$P6_3/mmc$	$P6_3/mmc$
a/Å	16.7782(18)	16.745(3)	16.7722(6)
c/Å	15.087(2)	15.088(3)	15.0089(7)
V/Å^3	3678.1(10)	3663.7(14)	3656.4(3)
Z	2	2	2
ρ_{calc} (g cm^{-3})	0.892	0.896	0.898
μ (mm^{-1})	5.041	5.065	0.629
R_1^a (I > 2σ)	0.0456	0.0388	0.0414
wR_2^b (all data)	0.1314	0.0999	0.1391
S	1.033	1.044	1.077

$^aR_1 = \Sigma||F_o| - |F_c||/\Sigma|F_o|$. $^bwR_2 = [\Sigma w(F_o^2 - F_c^2)^2/\Sigma w(F_o^2)^2]^{1/2}$.

MIL-88B-tpt[c], MIL-88B-tpt[d] and MIL-88B-tpt[e] were obtained *ex-situ* with excess 4-pyCN, *in-situ* with excess 4-pyCN, and *in-situ* without excess 4-pyCN, respectively.

Supplementary Table 1. (Continued)

Complex	4-pyCN@MIL-88B	3-pyCN@MIL-88B
Formula	$C_{28}H_{15}O_{14}N_2FFe_3$	$C_{42}H_{24}O_{13}N_6Fe_3$
Formula weight	789.97	988.22
Temperature (K)	361(2)	328(2)
Crystal system	Hexagonal	Hexagonal
Space group	$P6_3/mmc$	$P6_3/mmc$
a/Å	16.490(4)	15.9931(16)
c/Å	15.356(5)	15.9926(10)
V/Å^3	3616(2)	3542.5(7)
Z	2	2
ρ_{calc} (g cm^{-3})	0.726	0.926
μ (mm^{-1})	5.065	5.238
R_1^a (I > 2σ)	0.1268	0.1435
wR_2^b (all data)	0.2515	0.2906
S	1.042	1.057

$^aR_1 = \Sigma||F_o| - |F_c||/\Sigma|F_o|$. $^bwR_2 = [\Sigma w(F_o^2 - F_c^2)^2/\Sigma w(F_o^2)^2]^{1/2}$.

Supplementary Table 2. Atomic information of the final refinement results of the single-crystal X-ray diffraction data.

MIL-88B

Atom	*x*	*y*	*z*	*Relative Occupancy*	*Ueq.*
Fe1	0.25640(4)	0.51279(7)	3/4	1	0.0309(5)
O1	0.3460(2)	0.4995(2)	0.66609(16)	1	0.0535(9)
O2	1/3	2/3	3/4	1	0.0311(15)
O3	0.17632(19)	0.3526(4)	3/4	0.7534	0.0549(13)
F1	0.17632(19)	0.3526(4)	3/4	0.2466	0.0549(13)
C1	0.43633(19)	0.56367(19)	0.6392(3)	1	0.0422(12)
C2	0.4698(2)	0.5302(2)	0.5674(3)	1	0.0451(12)
C3	0.4015(3)	0.4318(4)	0.5336(3)	1	0.0638(13)

4-pyCN@MIL-88B

Atom	*x*	*y*	*z*	*Relative Occupancy*	*Ueq.*
Fe1	0.26663(7)	0.53327(15)	3/4	1	0.0716(9)
O1	0.3456(3)	0.5189(3)	0.6565(3)	1	0.0924(17)
O2	1/3	2/3	3/4	1	0.067(4)
O3	0.1922(4)	0.3844(8)	3/4	0.4200	0.142(5)
F1	0.1922(4)	0.3844(8)	3/4	0.2466	0.142(5)
N1	0.1922(4)	0.3844(8)	3/4	1/3	0.142(5)
N2	0	0	3/4	1	0.196(9)
C1	0.4274(3)	0.5726(3)	0.6309(7)	1	0.085(3)
C2	0.4651(3)	0.5349(3)	0.5626(7)	1	0.083(3)
C3	0.4096(6)	0.4477(6)	0.5309(6)	1	0.122(3)
C4	0.2391(10)	0.3360(12)	3/4	1/12	0.143(7)
C5	0.1921(15)	0.2397(12)	3/4	1/12	0.152(8)
C4'	0.1682(5)	0.3363(11)	0.6737(8)	1/12	0.147(6)
C5'	0.1198(5)	0.2396(10)	0.6721(8)	1/12	0.155(7)
C6	0.0953(6)	0.1906(12)	3/4	1/3	0.163(7)
C7	0.0424(6)	0.0849(12)	3/4	1/3	0.182(8)

3-pyCN@MIL-88B

Atom	*x*	*y*	*z*	*Relative Occupancy*	*Ueq.*
Fe1	0.26467(15)	0.5293(3)	3/4	1	0.0768(14)
O1	0.3452(6)	0.5146(6)	0.6603(4)	1	0.092(3)
O2	1/3	2/3	3/4	1	0.053(5)
C1	0.4289(6)	0.5711(6)	0.6335(8)	1	0.086(6)
C2	0.4653(6)	0.5347(6)	0.5630(8)	1	0.080(5)
C3	0.4089(10)	0.4435(10)	0.5327(7)	1	0.134(7)
N1	0.1882(6)	0.3764(13)	3/4	1	0.168(10)
C6	0.0878(6)	0.1756(13)	3/4	1	0.226(11)
C4	0.1905(9)	0.3263(12)	0.6824(7)	1/2	0.207(10)
C5	0.1412(10)	0.2259(12)	0.6803(6)	1/2	0.228(11)
C7	0.1450(18)	0.1748(18)	0.6075(10)	1/2	0.242(13)
N2	0.147(2)	0.133(2)	0.5505(13)	1/2	0.252(15)

MIL-88B-tpt

Atom	*x*	*y*	*z*	*Relative Occupancy*	*Ueq.*
Fe1	0.26797(2)	0.53595(4)	3/4	1	0.0265(3)
O1	0.34652(11)	0.52134(10)	0.65483(12)	1	0.0520(5)
O2	1/3	2/3	3/4	1	0.0265(9)
N1	0.19186(11)	0.3837(2)	3/4	1	0.0379(8)
N2	0.0941(3)	0.04704(14)	3/4	1	0.0680(14)
C1	0.42694(10)	0.57306(10)	0.6292(2)	1	0.0392(7)
C2	0.46478(11)	0.53522(11)	0.5622(2)	1	0.0455(8)
C3	0.41244(19)	0.44730(19)	0.5305(2)	1	0.0753(10)
C4	0.2354(2)	0.3350(2)	3/4	1	0.0569(9)
C5	0.1901(2)	0.2396(2)	3/4	1	0.0682(12)
C6	0.09596(16)	0.1919(3)	3/4	1	0.0583(14)
C7	0.04469(17)	0.0894(3)	3/4	1	0.0649(16)

MIL-88B-tpb

Atom	*x*	*y*	*z*	*Relative Occupancy*	*Ueq.*
Fe1	0.26445(3)	0.52891(5)	3/4	1	0.0330(4)
O1	0.34664(18)	0.52532(17)	0.6518(2)	1	0.0821(11)
O2	1/3	2/3	3/4	1	0.0221(12)
N1	0.19199(17)	0.3840(3)	3/4	1	0.0514(14)
C1	0.42527(18)	0.57473(18)	0.6262(4)	1	0.0618(14)
C2	0.4638(2)	0.5362(2)	0.5600(5)	1	0.0763(19)
C3	0.4131(3)	0.4491(3)	0.5298(5)	1	0.128(3)
C4	0.2315(5)	0.3369(5)	0.7749(6)	1/2	0.097(6)
C5	0.1840(5)	0.2415(5)	0.7702(16)	1/2	0.148(10)
C6	0.0973(2)	0.1945(5)	3/4	1	0.068(2)
C7	0.0473(3)	0.0947(5)	3/4	1	0.077(3)
C8	0.0937(6)	0.0468(3)	3/4	1	0.079(3)

MIL-88B-tpc

Atom	*x*	*y*	*z*	*Relative Occupancy*	*Ueq.*
Fe1	0.26415(3)	0.52829(7)	3/4	1	0.0435(5)
O1	0.3458(2)	0.5233(2)	0.8468(2)	1	0.0843(11)
O2	1/3	2/3	3/4	1	0.0300(13)
N1	0.1918(3)	0.3835(5)	3/4	1	0.095(2)
C1	0.42567(19)	0.57433(19)	0.8720(4)	1	0.0641(14)
C2	0.4641(2)	0.5359(2)	0.9399(4)	1	0.0719(16)
C3	0.4126(4)	0.4489(3)	0.9705(4)	1	0.110(2)
C4	0.2320(9)	0.3343(9)	3/4	3/4	0.136(3)
C5	0.1888(10)	0.2438(10)	3/4	3/4	0.154(4)
C4'	0.1680(6)	0.3360(12)	0.6710(11)	1/4	0.120(4)
C5'	0.1207(6)	0.2414(11)	0.6677(8)	1/4	0.138(4)
C6	0.0980(5)	0.1960(9)	3/4	1	0.150(4)
C7	0.0463(5)	0.0927(9)	0.774(2)	1/2	0.158(5)
C8	0.0864(12)	0.0432(6)	0.726(2)	1/2	0.146(6)

Supplementary Methods

Peak listing of spectral data for three trimmers.

tpt: ^{1}H NMR (400 MHz, DCl, 298 K, δ / ppm, Supplementary Figure 6): 7.34-7.35 (d, J = 4.0 Hz, 6H), 7.58-7.60 (d, J = 8.0 Hz, 6H); ^{13}C NMR (400 MHz, DCl, 298 K, δ / ppm, Supplementary Figure 7): 125.31, 140.76, 149.10, 167.90. IR (v/cm^{-1}, Supplementary Figure 4a): 3051.8, 3036.6, 1575.4, 1516.5, 1449.4, 1415.7, 1374.1, 1318.4, 1305.0, 1225.2, 1157.7, 1061.6, 1053.0, 992.7, 885.4, 844.5, 794.5, 666.6, 641.8, 503.9. ESI(+)-MS (calculated: 312.1, Supplementary Figure 5a): found: 313.0 ($[M+H]^+$).

tpb: ^{1}H NMR (400 MHz, DCl, 298 K, δ / ppm, Supplementary Figure 8): 6.73 (s, 3H), 6.75-6.76 (d, J = 4.0 Hz, 6H), 7.07-7.08 (d, J = 4.0 Hz, 6H). ^{13}C NMR (400 MHz, DCl, 298 K, δ / ppm, Supplementary Figure 9): 123.18, 129.51, 135.27, 140.16, 154.13. IR (v/cm^{-1}, Supplementary Figure 4b): 3037.1, 2924.7, 2854.0, 1633.9, 1590.4, 1550.2, 1501.0, 1402.5, 1384.5, 1325.6, 1193.3, 1072.8, 992.5, 964.4, 837.8, 814.3, 744.9, 611.7, 527.2. ESI(+)-MS (calculated: 309.1, Supplementary Figure 5b): found: 310.1 ($[M+H]^+$).

tpc: ^{1}H NMR (400 MHz, $CDCl_3$, 298 K, δ / ppm, Supplementary Figure 10): 1.67-1.77 (q, J = 12.0 Hz, 3H), 2.17-2.20 (d, J = 12.0 Hz, 3H), 2.93-2.99 (t, J = 12.0 Hz, 3H), 7.19-7.21 (d, J = 8.0 Hz, 6H), 8.53-8.54 (d, J = 4.0 Hz, 6H). ^{13}C NMR (400 MHz, $CDCl_3$, 298 K, δ / ppm, Supplementary Figure 11): 39.12, 43.15, 122.14, 150.00, 153.91. IR (v/cm^{-1}, Supplementary Figure 4c): 3054.2, 3020.3, 2987.5, 2941.2, 2912.1, 2893.4, 2863.1, 1947.9, 1703.6, 1600.7, 1594.4, 1553.4, 1495.3, 1452.0, 1438.5, 1412.8, 1373.7, 1317.6, 1222.3, 1112.8, 1072.6, 992.1, 965.2, 947.7, 869.5, 830.4, 817.7, 801.5, 746.3, 638.2, 605.3, 563.4, 542.8, 455.4, 417.9. ESI(+)-MS (calculated: 315.2, Supplementary Figure 5c): found: 316.1 ($[M+H]^+$).
